# Multi-component Microcapsules Derived Spatiotemporal Sonodynamic Reinforcing Therapy against Rheumatoid Arthritis

**DOI:** 10.34133/research.0844

**Published:** 2025-09-18

**Authors:** Danqing Huang, Min Nie, Rui Liu, Chang Xu, Jingjing Gan, Yuanjin Zhao, Lingyun Sun

**Affiliations:** ^1^ Department of Rheumatology and Immunology, Institute of Translational Medicine, Nanjing Drum Tower Hospital, Affiliated Hospital of Medical School, Nanjing University, Nanjing 210002, China.; ^2^ Department of Rheumatology and Immunology, Nanjing Drum Tower Hospital Clinical College of Nanjing University of Chinese Medicine, Nanjing, Jiangsu 210008, China.; ^3^ Institute of Organoids on Chips Translational Research, Henan Academy of Sciences, Zhengzhou 450009, China.

## Abstract

Microcarriers have demonstrated substantial potential in therapeutic delivery for rheumatoid arthritis (RA) treatment. Herein, we propose a novel spatiotemporal ultrasound-responsive microcapsule carrier to reinforce curcumin-based sonodynamic therapy in treating RA. Based on microfluidic technology, microcapsules with a hydrogel shell loaded with curcumin and a perfluorocarbon core enriched with oxygen (CUR/O_2_-MCs) can be obtained. Ascribed to the liquid-to-gas transition property of perfluorocarbon and the sonosensitivity of curcumin, ultrasound stimulation can realize controlled oxygen release followed by sonodynamic reactive oxygen species production. We have demonstrated that the ultrasound-activated CUR/O_2_-MCs can elevate reactive oxygen species amounts in overproliferating fibroblast-like synoviocytes, subsequently inducing fibroblast-like synoviocyte apoptosis. In addition, the released curcumin and oxygen can bring about macrophage repolarization, antiangiogenesis, and hypoxia alleviation, which are also important in blocking RA progression. Thus, in collagen-induced arthritis rats, our spatiotemporal sonodynamic microcapsules can attenuate symptoms and reduce joint damage. These results indicate that our ultrasound-responsive multifunctional microcapsule is an ideal candidate for sonodynamic therapy enhancement and RA treatment.

## Introduction

Rheumatoid arthritis (RA) is a chronic autoimmune disease with high disability [1]. The early pathological changes in RA mainly include fibroblast-like synoviocyte (RA-FLS) overproliferation, pannus invasion, and inflammatory cell aggregation [2,3]. Clinically, pharmacotherapy serves as the primary approach to controlling RA symptoms at an early stage, including disease-modifying antirheumatic drugs, nonsteroidal anti-inflammatory drugs, and glucocorticoids [4]. Additionally, various Chinese medicines, such as curcumin, have been demonstrated to possess therapeutic efficacy [5]. Emerging therapeutic strategies for RA have emerged, including photodynamic synovectomy and sonodynamic therapy. However, challenges remain, such as the limited penetration capability of light, the selection of sonosensitizers, and the modes of administration [6–8]. To sustain optimal drug concentration and achieve long-term therapeutic outcomes, these drugs should be administrated systematically with a high dosage. Although symptoms can be alleviated, systemic side effects are unavoidable, and serious complications can even lead to patient mortality. Currently, particle-based delivery systems have garnered considerable attention for their potential to enhance medication effectiveness [9–13]. By encapsulating drugs into micro- or nanoparticles, the sustained drug release can realize long-term treatment through carrier degradation. However, challenges remain in controlling the release of active compounds to achieve ideal drug concentrations. Moreover, numerous drug–drug interactions can markedly amplify therapeutic effects, while the simplistic structure and composition of existing drug carriers constrain their potential efficacy. Therefore, new drug carriers featuring advanced structural designs and enhanced functionalities are highly anticipated.

**Fig. 1.**
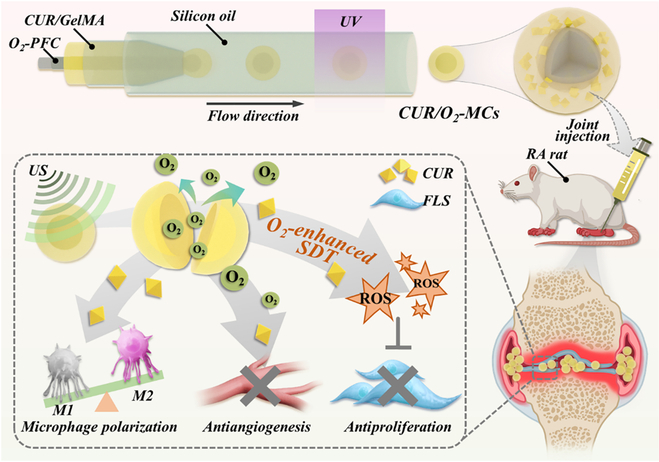
Schematic illustration of the curcumin-encapsulated core–shell microcapsules for rheumatoid arthritis (RA) treatment with ultrasound stimulation. CUR, curcumin; GelMA, gelatin methacryloyl; PFC, perfluorocarbon; UV, ultraviolet; CUR/O_2_-MCs, microcapsules with a hydrogel shell loaded with CUR and a PFC core enriched with oxygen; FLS, fibroblast-like synoviocyte; US, ultrasound; SDT, sonodynamic therapy; ROS, reactive oxygen species.

Herein, we propose a novel spatiotemporal ultrasound (US)-responsive microcapsule to reinforce curcumin treatment against RA, as illustrated in Fig. 1. US can transport energy remotely and efficiently through complex media, making it an ideal tool for internal excitation [14,15]. Combined with US-responsive components, numerous therapeutic strategies have been developed, such as sonodynamic therapy [16,17]. Coincidently, curcumin is a kind of sonosensitizer for sonodynamic therapy, which can catalyze oxygen in the surrounding environment to generate abundant reactive oxygen species (ROS) under specific US stimulation. Studies have shown that such ROS bursts can induce irreversible oxidative damage to abnormal cells and lead to cell apoptosis, showing a definite therapeutic effect in treating RA by eliminating RA-FLSs [6,7,18,19]. In addition, considering the consumption of a large amount of oxygen during sonodynamic therapy and the hypoxic microenvironment in RA joint cavities, additional oxygen supply is crucial in enhancing sonodynamic therapy effectiveness. In contrast, microfluidic technology is superior in fabricating microcarriers with sophisticated structures, allowing for loading of various active substances within their different regions [20–23]. Thus, constructing a microcarrier with specifically arranged multiple US-responsive substances via microfluidic technology is expected to reinforce sonodynamic therapy against RA.

In this study, we propose an oxygen- and curcumin-delivered spatiotemporal microcapsule by using microfluidics for US-assisted RA treatment. Attributed to the maneuverability of microfluidic technology, we designed a core–shell microcapsule with a hydrogel shell encapsulating the sonosensitizer curcumin and a core loaded with oxygen-enriched fluoro-nitrogen compound (CUR/O_2_-MC). It was demonstrated that CUR/O_2_-MCs showed outstanding US responsiveness in regulated curcumin release, controlled oxygen release, and enhanced ROS production. Additionally, we verified that US-stimulated CUR/O_2_-MCs can effectively elevate intracellular ROS and inhibit RA-FLS proliferation. In contrast, the released oxygen can reverse the hypoxic microenvironment; meanwhile, the released curcumin can significantly induce macrophage repolarization and inhibit angiogenesis, which is of great significance in inhibiting RA development. Based on these features, we further illustrated that after treating RA rats with intra-articularly injected CUR/O_2_-MCs and US irradiation, their diseased joints showed significantly alleviated symptoms and effectively inhibited bone erosion. These results indicated that out US-responsive multifunctional microcapsules can target progression factors and efficiently improve sonodynamic therapy efficacy in RA treatment.

## Results and Discussion

**Fig. 2.**
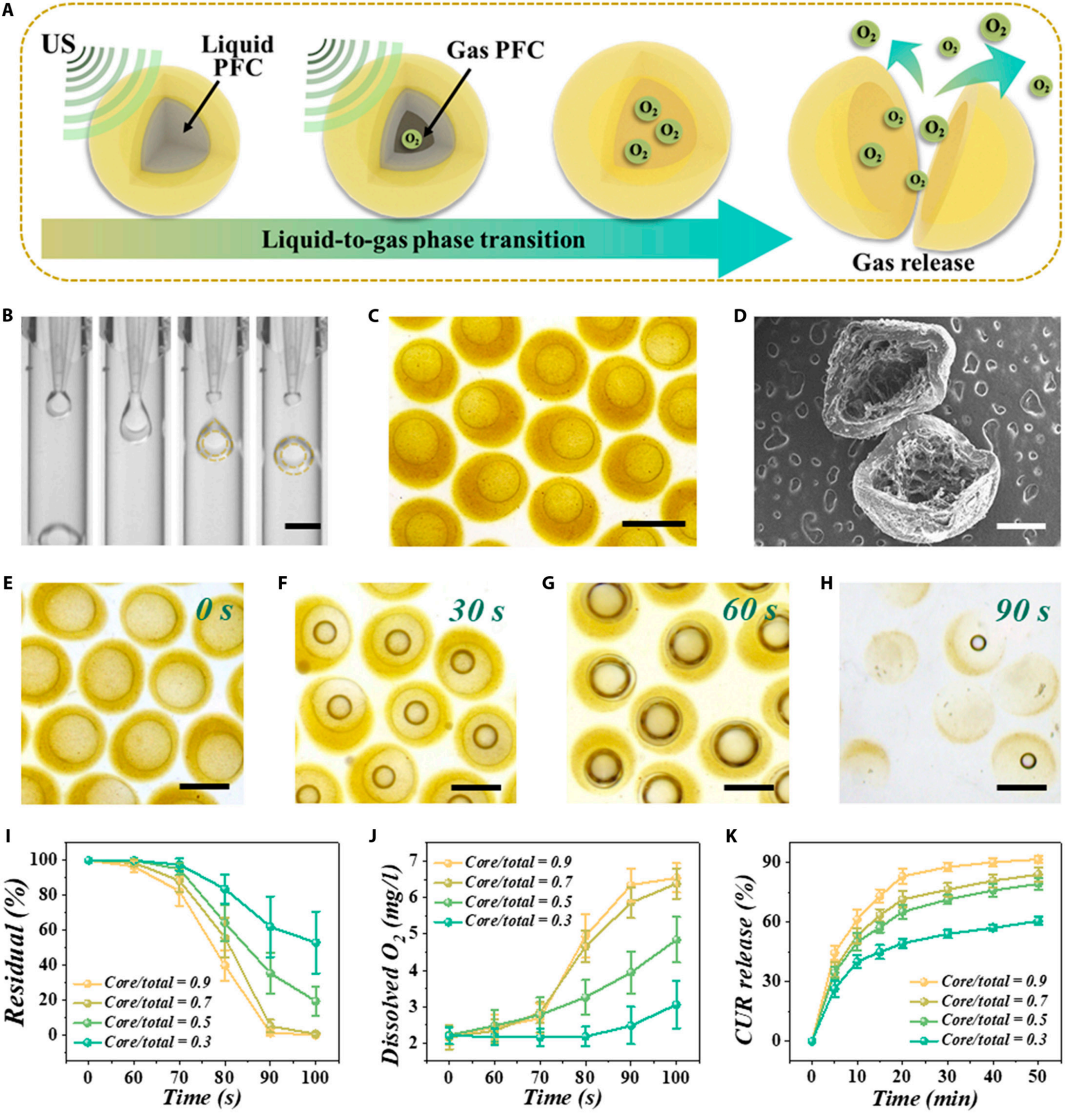
Fabrication of microcapsules and the US responsiveness. (A) Schematic diagram of the US-activated morphological transition. (B) High-speed camera photographs of the double-emulsion microfluidic fabrication process. (C) Stereomicroscopic image of the CUR/O_2_-MCs. (D) Scanning electron microscopy (SEM) image of a dissected microcapsule. (E to H) Morphology variation of microcapsules at 0- (E), 30- (F), 60- (G), and 90-s (H) US stimulation. (I) Proportions of intact microcapsules remaining with different core–total ratios after different US stimulation times (*n* = 5). (J) Concentrations of dissolved oxygen released by microcapsules with different core–total ratios after different US stimulation times (*n* = 5). (K) Curcumin release curves of microcapsules with different core–total ratios within 50 min (*n* = 5). Scale bars are 300 μm in (B) and (E) to (H), 200 μm in (C), and 100 μm in (D).

In this study, we fabricated core–shell microcapsules encapsulating US-responsive liquid perfluorocarbon (PFC) to realize controllable oxygen release via microfluidic technology (Fig. 2A). Since PFC is a substance that remains liquid and is stable only in a closed environment at normal temperature and pressure, the gentle and rapid material processing afforded by microfluidic technology is highly beneficial for preserving the liquid state of PFC. A double-emulsion microfluidic chip was made for the fabrication of microcapsules using capillaries and a glass slide (Fig. S1). The outermost, middle, and innermost channels were filled with silicon oil, curcumin-dispersed gelatin methacryloyl (GelMA) pregel solution, and oxygen-enriched liquid PFC, respectively. Due to the incompatibility between the hydrophilic GelMA solution and the oily PFC, the 2-phase liquid forms a laminar flow, followed by the formation of oil-in-water monodisperse droplets under the shearing force of the high-flow-rate silicone oil (Fig. 2B). After polymerization using ultraviolet light, microcapsules with a GelMA shell and a PFC core were obtained (Fig. 2C). The core–shell structure can be illustrated in a scanning electron microscopy image (Fig. 2D). Moreover, because the use of a microfluidic pump can fine-adjust the flow rate of liquid in each channel, we prepared a variety of microcapsules with different core–total ratios and investigated the relationship between flow rate and core–total ratio (Fig. S2).

Then, we investigated the US responsiveness of our PFC-core microcapsules. Liquid PFC is a fluorocarbon compound with a very low boiling point (55 °C), while US with certain power and intensity can cause hyperthermia via cavitation and the acoustothermal effect. According to our previous study, US with specific intensity power and central frequency can effectively increase the temperature of liquid PFC to its boiling point, thereby inducing a phase transition from liquid to gas. As shown in Fig. 2E, microcapsules with a liquid PFC core featured uniformity, monodispersity, and stability. Under the continuous US stimulation, a gas cavity appeared in the liquid PFC core and gradually expanded (Fig. 2F and G). Finally, after approximately 90-s US treatment, the microcapsules collapsed and the encapsulated gas was released (Fig. 2H). Furthermore, in order to explore the influence of shell thickness and core volume on US responsiveness, we prepared 4 kinds of microcapsules with different core–total ratios (0.9, 0.7, 0.5, and 0.3) by adjusting microfluidic parameters and calculated the number of microcapsules that had ruptured under continuous US stimulation. As illustrated in Fig. 2I, microcapsules with thinner shells (core/total = 0.9 and 0.7) almost all ruptured under US stimulation for about 90 to 100 s, while the microcapsules with thicker shells (core/total = 0.5 and 0.3) remained mostly unbroken. These results indicated that microcapsules with core–total ratios of 0.9 and 0.7 showed better US responsiveness.

Since PFC has a markedly higher oxygen dissolution capacity than water, PFC can be used as a blood substitute in clinical practice, reflecting its biosafety and oxygen-carrying capacity. During the fabrication process of microcapsules, PFC was enriched with oxygen and then was encapsulated into a hydrogel shell. By detecting the dissolved oxygen in the experimental liquid environment, we found that, consistent with the microcapsule rupture ratio, the microcapsule groups with a thinner shell (core/total = 0.9 and 0.7) had a higher dissolved oxygen content (Fig. 2J). Additionally, we measured the oxygen release without US stimulation. Results showed that the microcapsule groups with a thicker shell had a relatively higher retention of encapsulated oxygen, indicating that the lower core–total ratio contributed to higher stability (Fig. S3A). These results indicated that microcapsules with PFC core can realize US-triggered oxygen release. By further loading the sonosensitizer curcumin into the hydrogel shell, we investigated the drug release kinetics, encapsulation efficiency, and loading capacity of microcapsules with different core–total ratios (Fig. 2K and Figs. S3B and S4). The GelMA pregel solution used for the microfluidic fabrication process was a curcumin suspension. It can be concluded that microcapsules with core–total ratios of 0.9, 0.7, and 0.5 showed higher dug release efficiency, while microcapsules with a core–total ratio of 0.9 showed a limited drug-loading capacity. Therefore, we optimized 0.7 core–total ratio microcapsules for further investigation. According to the aforementioned microfluidic parameters, we applied a 20 μl/min PFC flow rate, an 80 μl/min GelMA flow rate, and a 500 μl/min silicon oil flow rate to fabricate microcapsules. We further calculated the core and microcapsule diameters of the final obtained microcapsules, and this diameter allows them to be administered via clinically used injection needles (Fig. S5). Considering the various conditions before application and in vivo, the stability of these microcapsules was evaluated. As illustrated in Fig. S6, these CUR/O_2_-MCs exhibited good stability in solutions of varying pH, while their stability deteriorated as the temperature increased, likely due to the very low boiling point of PFC.

**Fig. 3.**
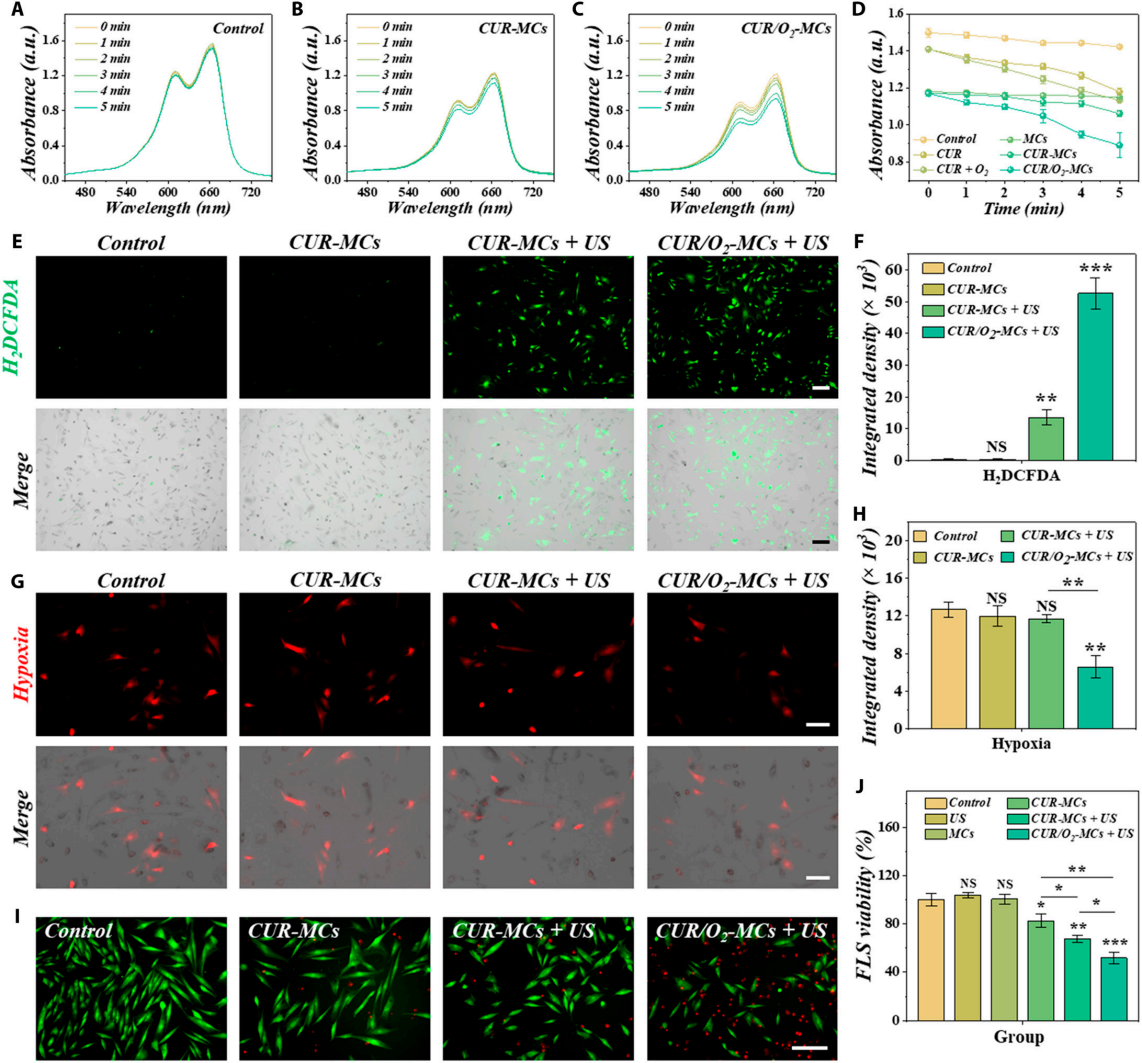
Analysis of curcumin’s ROS production and RA-FLS cytotoxicity. (A to C) Methylene blue (MB) degradation analysis of curcumin with and without oxygen exposure. (D) MB absorbance peaks of different groups at different time intervals (*n* = 3). (E and F) Fluorescent images (E) and quantified analysis (F) of intracellular ROS staining using 2′,7′-dichlorodihydrofluorescein diacetate (H_2_DCFDA) (*n* = 3). (G and H) Fluorescent images (G) and quantified analysis (H) of hypoxic conditions using a hypoxia red detection reagent (*n* = 3). (I) Live and dead staining of RA-FLSs. (J) Cell Counting Kit-8 (CCK-8) results of RA-FLSs (*n* = 5). Scale bars are 200 μm in (E), (G), and (I). *P < 0.05; **P < 0.01; ***P < 0.001; NS, not significant.

As a widely applied Chinese medicine, curcumin is also a sonosensitizer that has shown promising potential in sonodynamic therapy. We first evaluated its US-stimulated ROS production using methylene blue (MB). According to various studies, the more ROS are produced, the more MB is degraded. Compared with the control group, the peak absorbance of MB decreased significantly in the CUR group and CUR-O_2_ group, indicating that the US sensitivity of curcumin and the exogenously provided oxygen can improve ROS production efficiency (Fig. 3A and Fig. S7). Furthermore, we evaluated the sonosensitivity of the curcumin encapsulated by hydrogel (Fig. 3B and C). Results showed that under the US excitement, CUR-MCs produced certain ROS, with CUR/O_2_-MCs producing significantly more ROS (Fig. 3D).

Before cytotoxicity evaluation on RA-FLSs, we first verified the biocompatibility of the empty-loaded microcapsules and US on healthy FLSs and RA-FLSs (Fig. S8). Results showed that the GelMA hydrogel, PFC, and US would not harm cells. We then further optimized the dose of curcumin, to ensure that the proliferation of RA-FLSs can be efficiently inhibited under curcumin-based sonodynamic therapy without damaging normal cells (Figs. S9 and S10). By staining intracellular ROS with 2′,7′-dichlorodihydrofluorescein diacetate (H_2_DCFDA) (green fluorescence), it can be demonstrated that the RA-FLSs treated with US-excited microcapsules emitted strong green fluorescence (Fig. 3E and Fig. S11). Additionally, the content of ROS in the RA-FLSs treated with CUR/O_2_-MCs was significantly higher, suggesting that exogenous oxygen could enhance ROS production (Fig. 3F). Moreover, we demonstrated a markedly alleviated hypoxic condition of the RA-FLSs treated with CUR/O_2_-MCs (Fig. 3G and H and Fig. S12). Finally, it was verified that US-triggered CUR/O_2_-MCs can significantly induce RA-FLS apoptosis (Fig. 3I and J and Fig. S13). Furthermore, the proliferation inhibition of sonodynamic therapy on RA-FLSs was verified through cell migration experiments (Fig. S14). These results indicated that our curcumin- and oxygen-loaded microcapsules can efficiently elevate ROS production, alleviate hypoxic conditions, and induce RA-FLS apoptosis without harming healthy cells, paving way for its application in RA treatment.

**Fig. 4.**
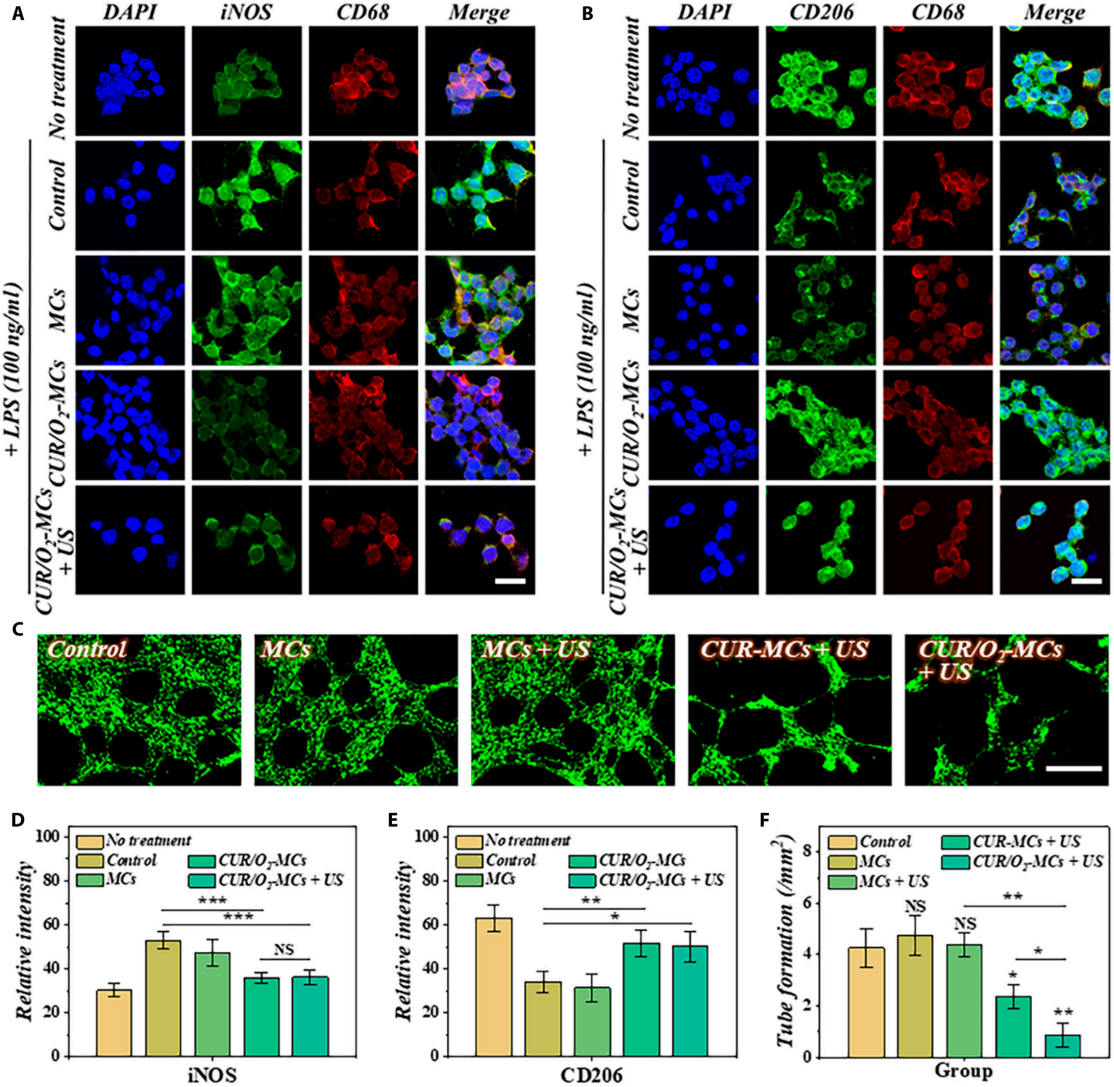
Analysis of CUR/O_2_-MCs’ effect on macrophage polarization and angiogenesis. (A) Immunofluorescence staining of 4′,6-diamidino-2-phenylindole (DAPI; blue), inducible nitric oxide synthase (iNOS; green), and CD68 (red) on macrophages. (B) Immunofluorescence staining of DAPI (blue), CD206 (green), and CD68 (red) on macrophages. (C) Tube formation analysis of human umbilical vein endothelial cells (HUVECs). (D and E) Quantified analysis of iNOS (D) and CD206 (E) fluorescent intensity (*n* = 5). (F) Tube formation analyses (*n* = 3). Scale bars are 100 μm in (A) and (B), and 500 μm in (C). *P < 0.05; **P < 0.01; ***P < 0.001; NS, not significant.

There are many pathogenic factors participating in the occurrence and development of RA, such as the polarization of macrophages, compensatory proliferation of new vessels, and formation of a hypoxic environment. These factors affect each other and jointly promote the progression of RA. Curcumin has a variety of biological activities, including anti-inflammatory, antioxidant, anticancer, antibacterial ones. Multiple studies have confirmed that curcumin may promote the polarization of macrophages toward the M2 phenotype through mechanisms such as activating the PPAR-γ pathway or inhibiting the NF-κB and PI3K–AKT/STAT3 pathways [24–27]. Therefore, we investigated the effect of curcumin-loaded microcapsules on the pathogenic factors of RA. Using murine RAW 264.7 macrophages, we evaluated the effects of CUR/O_2_-MCs in macrophage polarization. As illustrated in Fig. 4A and B, macrophages co-cultured with CUR/O_2_-MCs effectively repolarized from an inflammatory to an anti-inflammatory type (Fig. 4D and E). Notably, we observed a reduction in macrophage numbers when US treatment was combined. We therefore further assessed macrophage activity across different treatment groups. Our findings revealed that sonodynamic therapy diminishes macrophage activity (Fig. S15A). However, overall, the sustained release of anti-inflammatory curcumin drives the polarization of macrophages from the M1 to the M2 phenotype, which remains beneficial for RA treatment. Consistent results can be concluded through pro-inflammatory cytokines, including tumor necrosis factor-α (TNF-α) and interleukin (IL)-6, and anti-inflammatory cytokines, including IL-10 and arginase-1, detected from the supernatant via enzyme-linked immunosorbent assay. Macrophages treated with CUR/O_2_-MCs showed a significantly decreased TNF-α and IL-6 expression levels, with a markedly increased IL-10 and arginase-1 expression levels, compared to the control group and MCs group (Fig. S16). Given that the compensatory proliferation of neovascularization and the formation of pannus are also critical factors driving the progression of RA, we further investigated the impact of CUR/O_2_-MCs and sonodynamic therapy on vascular endothelial cells. Results showed that the application of hydrogel microcapsules and US would not affect the viability and tube formation of human umbilical vein endothelial cells (HUVECs). However, the combination of curcumin and US significantly reduced the number of cells, particularly when additional oxygen was supplied. It was indicated that after treatment with CUR/O_2_-MCs and US, the proliferation of HUVECs can be significantly inhibited (Fig. 4C and F and Fig. S15B). These results indicated that the curcumin- and oxygen-loaded platform can effectively induce macrophage polarization and inhibit the proliferation of vascular endothelial cells, facilitating RA treatment.

**Fig. 5.**
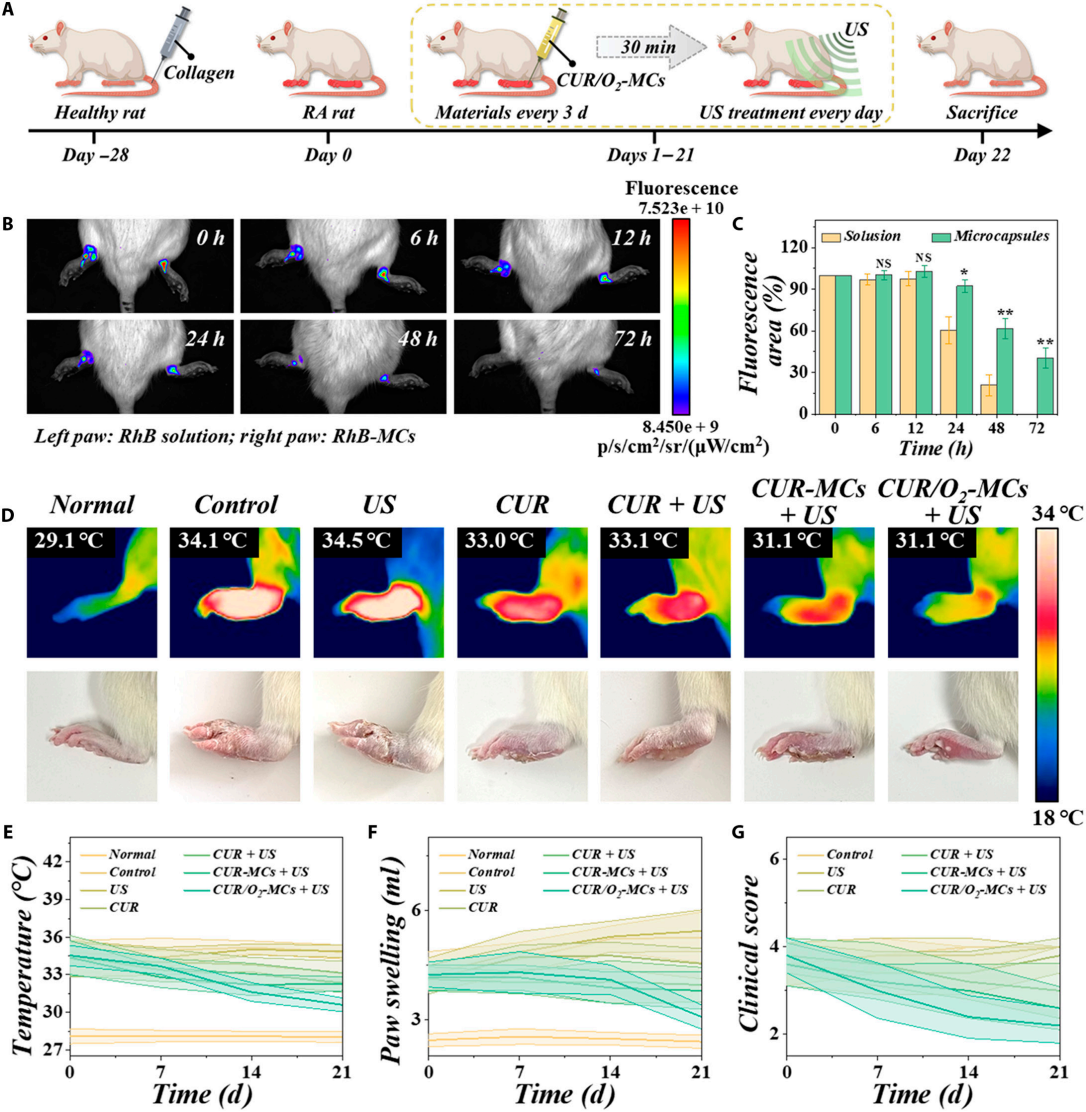
Therapeutic efficacy of CUR/O_2_-MCs on the collagen-induced arthritis (CIA) rat model. (A) Schematic illustration of CIA rat establishment and treatment. (B) In vivo imaging showed the drug localization in the rat’s articular cavity at different time intervals. (C) Relative fluorescence area of drugs in the rat’s articular cavity (*n* = 3). (D) Digital and thermographic photos of left paws from different groups on day 14. (E to G) Variation of paw temperatures, paw volumes, and clinical scores at treatment days 0, 7, 14, and 21 (*n* = 5). RhB, rhodamine B. *P < 0.05; **P < 0.01; NS, not significant.

To elucidate the therapeutic efficacy of CUR/O_2_-MC-affiliated sonodynamic therapy on treating RA, we established a collagen-induced arthritis (CIA) mouse model. After injecting collagen and incomplete adjuvant emulsion into the tail root of the rats, we confirmed the successful establishment of the CIA model by evaluating the clinical scores of the rats and started animal experiments on day 28 (Fig. 5A). The CIA rats were randomized into 6 groups, i.e., control, US, CUR, CUR + US, CUR-MCs + US, and CUR/O_2_-MCs groups. For rats in the treatment group, after anesthesia, drugs were administered via intra-articular injection. Before administration, the skin at the injection site was thoroughly disinfected. Following injection, sterile cotton was used to gently press the area for several minutes to prevent bleeding and drug leakage. During the subsequent experimental process, the skin at the injection site was monitored, and no obvious ulceration or infection was observed. In order to ensure the drug sustaining concentration in the joint cavity and optimize the administration interval, we first injected the small-molecule fluorescent drug rhodamine B into the joint cavity of rats. By injecting rhodamine B solution into the left articular cavity and rhodamine B-encapsulated microcapsules into the right articular cavity, we can observe by using a small-animal fluorescence imaging system that the drugs can accumulate in place for at least 3 d (Fig. 5B). Additionally, ascribed to the sustained release of hydrogel microcapsules, the concentration and retention of drugs in the right articular cavity was significantly longer (Fig. 5C).

As arthritis develops and progresses, the joints of rats show hyperthermia and swelling. By recording changes in the temperature and volume of the rats’ hind paws during treatment, we found that the temperature and swelling of the paws from the CUR, CUR + US, CUR-MCs + US, and CUR/O_2_-MCs+ US groups were significantly reduced comparing with those of the control group (Fig. 5D). Especially in the CUR/O_2_-MCs + US group, the temperature of the paws dropped significantly, and only the ankle joint remained slightly hyperthermic (Fig. 5E). In addition, by observing the swelling of the hind paws and the walking gait of the rats, we found that the joint movement of the rats in the CUR/O_2_-MCs+ US group was more flexible (Fig. 5F). We also evaluated the clinical scores of the rats from different groups, indicating the ideal recovery of arthritis (Fig. 5G). After the 22-d treatment, the paws and joints, as well as the main organs, were harvested for further analyses (Figs. S17 and S18).

**Fig. 6.**
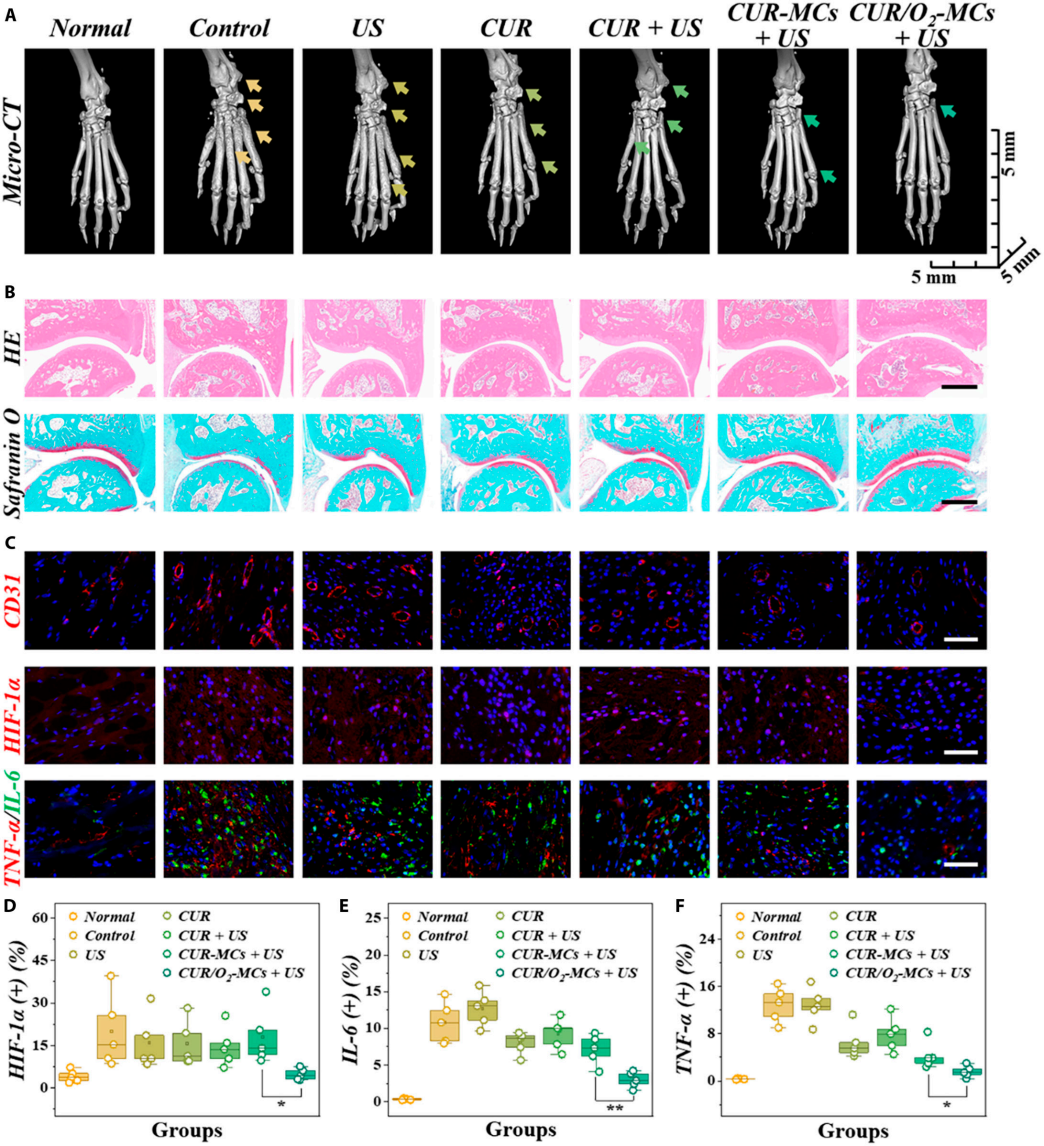
Microcomputed tomography (micro-CT) and histological evaluation of CUR/O_2_-MCs’ therapeutic efficacy. (A) Micro-CT images of paws (arrows: bone damage). (B) Hematein and eosin (HE) and safranin O staining images of the joints from different groups. (C) Immunohistochemical staining images of the synovial tissue from different groups. (D to F) Fluorescence quantitative results of hypoxia-inducible factor-1α (HIF-1α) (D), interleukin-6 (IL-6) (E), and tumor necrosis factor-α (TNF-α) (F) (*n* = 5). Scale bars are 100 μm in (B) and 50 μm in (C). *P < 0.05; **P < 0.01.

The bone erosion was further evaluated via microcomputed tomography (micro-CT). As shown in Fig. 6A and Fig. S19, severe bone damage can be observed in the control and US groups, while markedly alleviated bone erosion can be noticed in the CUR/O_2_-MCs + US group. Consistent with the micro-CT images, the bone and trabecular (Tb) parameters of the joints from different groups also verified the reduced bone injury in the CUR/O_2_-MCs + US group, according to the higher bone volume, Tb number, Tb thickness, Tb connection density, and degree of anisotropy (Fig. S20). In hematein and eosin staining images, it can be noticed that compared with the normal group, severe cartilage damage and abnormal synovial tissue proliferation occurred in the control and US groups (Fig. 6B). However, the curcumin-treated joints showed relatively intact bones and cartilages and reduced histological scores (Fig. S21A), especially in the CUR/O_2_-MCs + US group. Consistently, the joints from the control and US group showed significant cartilage loss, while the pathological features in the CUR-MCs + US and CUR/O_2_-MCs + US groups were minimal.

Furthermore, we conducted immunofluorescence staining of the synovial tissue in the joints to investigate their angiogenesis (CD31, red fluorescence), hypoxic conditions (HIF-1α, red fluorescence), and inflammatory microenvironment (TNF-α, red fluorescence; IL-6, green fluorescence). As illustrated in Fig. 6C, curcumin-based sonodynamic therapy can effectively inhibit angiogenesis in synovial tissue. Compared with that of the CUR-MCs + US group, vascularity was further inhibited in the CUR/O_2_-MCs + US group, which might be due to the external supply of oxygen reinforcing sonodynamic therapy (Figs. S21B and S22). As the overproliferation of synovial tissue and vascularity would lead to excessive consumption of oxygen, the resulting hypoxic microenvironment would further promote the pathological progression of RA. Attributed to the oxygen delivered by the microcapsules, the hypoxic condition was alleviated in the joints in the CUR/O_2_-MCs + US group, which can be observed through hypoxia-inducible factor-1α (HIF-1α) immunohistochemistry staining (Fig. 6C and D and Fig. S23). Compared with that in the other RA joints, the HIF-1α was down-regulated to a nearly normal level in the CUR/O_2_-MCs + US group. Additionally, the inflammation condition in joints was evaluated through immunohistochemistry staining of TNF-α and IL-6. It can be observed that the curcumin-treated cases showed a certain reduction of TNF-α and IL-6 expression. In particular, the expression of these inflammatory factors was significantly reduced in the CUR/O_2_-MCs + US group, approaching normal levels (Fig. 6E and F and Fig. S24). These results indicated the certain therapeutic efficiency of curcumin-based sonodynamic therapy in treating RA and the pivotal role of the externally delivered oxygen.

## Conclusion

In conclusion, to reinforce sonodynamic therapy efficacy in treating RA, we developed a novel spatiotemporal US-responsive microcapsule carrier, codelivering the sonosensitizer curcumin and oxygen to induce diseased cell apoptosis in arthritis joints. Curcumin is a kind of natural Chinese medicine derived from turmeric, which has remarkable effects in anti-inflammatory and bactericidal aspects. Studies have verified that curcumin also features excellent sonosensitivity, generating ROS under certain US stimuli. To date, photodynamic and sonodynamic therapy has yielded attention in the biomedical field, and photo/sonosensitizers such as porphyrins, xanthenes, and piezoelectric materials have been widely explored [28,29]. Clinical trials of photodynamic therapy for RA have also been carried out and achieved ideal therapeutic effects [8]. Compared with traditional therapies, sonodynamic therapy, which uses US for external stimulation, allows minimally invasive, remote, and targeted treatment. Most importantly, since sonodynamic therapy primarily induces apoptosis of diseased cells through acute oxidative damage, it effectively circumvents the development of drug resistance and reduces the risk of disease recurrence. Previous studies have verified the applicability of using sonodynamic therapy for RA treatment [6,7]. However, the systematic administration of drugs still has limited targeted accumulation in diseased joints. Given that intra-articular injection is already a well-established clinical treatment approach, we hypothesize that developing a multi-drug-loaded carrier for intra-articular administration, when combined with sonodynamic therapy, could enhance intra-articular drug concentrations and improve therapeutic efficacy with minimally invasiveness. Various research studies have applied curcumin as a sonosensitizer to realize anti-tumor, antibacterial, and antiatherosclerosis sonodynamic therapy. However, the application of curcumin-based sonodynamic therapy for RA treatment has not been explored. Additionally, stimuli-responsive hydrogels stand out as a key advancement in this field, offering remarkable precision in spatiotemporal regulation and strong biocompatibility, making them ideal for targeted drug delivery [30]. On the other hand, microfluidic technology is an emerging fabrication method in the biomedical field, including drug delivery and bioengineering [31–34]. In this study, we not only confirmed that US-activated curcumin can induce the apoptosis of RA-FLSs by producing a large number of ROS but also realized codelivery of oxygen through the structured design of the sonosensitizer carrier to solve the problem of the consumption of a large amount of oxygen in the process of sonodynamic therapy.

Ascribing to the maneuverability and flexibility of microfluidic technology, we designed and fabricated a core–shell structure microcarrier via a double-emulsion microfluidic chip. To obtain stable oxygen encapsulation and efficient delivery, we applied liquid PFC as an oxygen carrier and encapsulated it into the microcarriers as cores. With the passive release of curcumin, ultrasonic stimulation with specific parameters can not only activate curcumin to produce ROS but also trigger the liquid PFC’s transition to gas to release oxygen. The released oxygen provides extra raw materials for ROS production in the sonodynamic therapy process; more importantly, it alleviates the hypoxic environment in the RA joint cavity and further enhances the anti-inflammatory effect of curcumin and antiangiogenesis effect of sonodynamic therapy. Furthermore, based on the rat CIA model, we confirmed that the spatiotemporal US-responsive microcapsule carriers can enhance the efficacy of sonodynamic therapy in RA, effectively reduce inflammation, inhibit synovial tissue overproliferation, and reduce bone and cartilage injury. These results indicated that our US-responsive multifunctional microcapsule is an ideal candidate for sonodynamic therapy enhancement and RA treatment.

## Materials and Methods

### Fabrication of microcapsules

The double-emulsion microfluidic chip was constructed with a glass slide and 3 coaxially assembled capillaries. A capillary with a 580-μm inner diameter was used as the outermost channel, referred to as the collection tube. Silicon oil was applied as the outer phase, GelMA pregel solution (7.0 wt.% GelMA, 0.1 vol.% 2-hydroxy-2-methylpropiophenone, and 0.05 M curcumin) was applied as the middle phase, and PFC enriched with oxygen (40 to 50 mg/l dissolved oxygen) was injected into the inner channel. By using 3 syringe pumps, silicon oil, curcumin–GelMA solution, and oxygen–PFC were pumped through the outer, middle, and inner channels, respectively. During the formation of the oil–water–oil emulsion within the capillary, ultraviolet light was applied to continuously illuminate the collection tube to polymerize GelMA. A petri dish filled with silicon oil was applied to collect the resultant microcapsules. The collected microcapsules were washed with phosphate-buffered saline (PBS) 3 times to remove the silicon oil and stored at room temperature in the dark before use.

### Characterization of microcapsules

During the microfluidic fabrication process, the flow rate of silicon oil (outer phase), curcumin–GelMA solution (middle phase), and oxygen–PFC (inner phase) was regulated as follows: adjusting the flow rate of the middle phase from 300 to 600 μl/min while keeping the inner and outer phases at 20 and 500 μl/min, respectively; adjusting the flow rate of the outer phase from 300 to 600 μl/min while keeping the inner and middle phases at 20 and 80 μl/min, respectively; and adjusting the flow rate of the inner phase from 5 to 35 μl/min while keeping the middle and outer phases at 80 and 500 μl/min, respectively. The obtained microcapsules were observed and pictured with a stereomicroscope. The core and microcapsule diameters were measured with the ImageJ software. A microcapsule was freeze-dried and cut open, and the core–shell structure was observed by scanning electron microscopy. The drug release characterization was conducted in PBS with 5% Tween 80 (Tween–PBS) to better simulate the in vivo conditions. The weight of curcumin initially dispersed into GelMA is referred to as *m*_1_, the weight of curcumin loaded into microcapsules is referred to as *m*_2_, and the whole weight of microcapsules is referred to as *m*_3_. We calculated encapsulation efficiency (%) by dividing *m*_1_ by *m*_2_ and loading capacity (%) by dividing *m*_3_ by *m*_2_.

### Ultrasonic responsiveness evaluation

A US transducer was applied to stimulate the microcapsules. Microcapsules were initially placed in 2 ml of Tween–PBS in a petri dish at room temperature, with US gel applied to the outside bottom of the petri dish. Then, US was applied to the bottom, and the microcapsules were observed and pictured with a stereomicroscope. To evaluate the US-triggered oxygen release capacity, approximately 300 microcapsules were immersed in 2 ml of Tween–PBS in a centrifuge tube. The dissolved oxygen was measured using a dissolved oxygen meter.

### MB degradation analysis

According to previous research and other studies, we confirmed ROS production by detecting MB degradation. To avoid the error caused by the material, we set up 6 experimental groups: control group (0.001 wt.% MB in 2 ml of Tween–PBS), MCs group (0.001 wt.% MB and ≈300 microcapsules in 2 ml of Tween–PBS), CUR group (0.001 wt.% MB and 40 μM curcumin in 2 ml of Tween–PBS), CUR + O_2_ group (0.001 wt.% MB, 40 μM CUR, and 1 μl of oxygen-enriched PFC in 2 ml of Tween–PBS), CUR-MCs group (0.001 wt.% MB and ≈100 CUR-MCs in 2 ml of Tween–PBS), and CUR/O_2_-MCs (0.001 wt.% MB and ≈100 CUR/O_2_-MCs in 2 ml of Tween–PBS). After US stimulation, 100 μl of the supernatant was taken and the absorption peak curves were measured using a microplate reader at 664 nm.

### Intracellular ROS detection and RA-FLS viability analysis

H_2_DCFDA was applied to measure the intracellular ROS level. RA-FLSs were divided into the control group (nontreatment), CUR-MCs group (co-culture with CUR-MCs for 30 min, ≈100 CUR-MCs), CUR-MCs + US group (co-culture with CUR-MCs for 30 min followed by 2-min US treatment, ≈100 CUR-MCs), and CUR/O_2_-MCs + US group (co-culture with CUR/O_2_-MCs for 30 min followed by 2-min US treatment, ≈100 CUR/O_2_-MCs). Immediately after treatment, the medium and microcapsules were dumped, and H_2_DCFDA (10 μM in PBS) was added. After incubating in the dark for 30 min, green fluorescence channel images and bright-field images were taken. For cell viability analysis, 4 groups of RA-FLSs were set the same as those in the ROS-detecting experiment. After different treatments, calcein-AM and propidium iodide were added after 24-h co-culture. After incubation in the dark for 20 min, green and red fluorescence channel images were taken.

### Macrophage polarization analysis

Murine RAW 264.7 macrophages were firstly pre-treated with lipopolysaccharides. Then, the lipopolysaccharide-treated macrophages were divided into the control group (nontreatment), MCs group (co-culture with empty-loaded MCs, ≈100 empty-loaded MCs), CUR/O_2_-MCs group (co-culture with CUR/O_2_-MCs, ≈100 CUR/O_2_-MCs), and CUR/O_2_-MCs + US group (co-culture with CUR/O_2_-MCs for 30 min followed by 2-min US treatment, ≈100 CUR/O_2_-MCs). After 24-h co-culture, the macrophages were stained with CD68 (pan-macrophage marker), inducible nitric oxide synthase (inflammatory marker), and CD206 (anti-inflammatory marker), followed by confocal laser scanning microscope recording.

### Tube formation analysis

HUVECs cultured on Matrigel were divided into the control group (nontreatment), MCs group (co-culture with empty-loaded MCs, ≈100 empty-loaded MCs), MCs + US group (co-culture with empty-loaded MCs for 30 min followed by 2-min US treatment, ≈100 empty-loaded MCs), CUR-MCs + US group (co-culture with CUR-MCs for 30 min followed by 2-min US treatment, ≈100 CUR-MCs), and CUR/O_2_-MCs + US group (co-culture with CUR/O_2_-MCs for 30 min followed by 2-min US treatment, ≈100 CUR/O_2_-MCs). After 12-h co-culture, the HUVECs were stained with calcein-AM and green fluorescence channel images were taken.

### CIA rat establishment and animal experiments

Thirty male Sprague–Dawley rats were selected to establish the CIA rat model. After anesthetizing, an emulsion containing porcine type II collagen was injected into the tail root of the rats. Arthritis symptoms were observed 3 to 4 weeks after injection. When the clinical scores of the CIA rats reached 4, they were divided into 6 groups, namely, the control (left untreated), US, CUR, CUR + US, CUR-MCs + US, and CUR/O_2_-MCs + US groups. Materials were injected into the footpad and ankles every 3 d with PBS (100 μl per rat in the US group), curcumin solution (0.05 mg in 100 μl of PBS per rat in the CUR and CUR + US groups), CUR-MCs (≈200 CUR-MCs in 100 μl of PBS per rat in the CUR-MCs + US group), and CUR/O_2_-MCs (≈200 CUR/O_2_-MCs in 100 μl of PBS per rat in the CUR/O_2_-MCs + US group), respectively. US treatment was applied to the footpad and ankles every day. Rats were sacrificed on day 22; the hind legs and main organs were harvested for further analyses.

## Data Availability

The data are freely available upon request.
