# Supplementary material for: Multi-component Microcapsules Derived Spatiotemporal Sonodynamic Reinforcing Therapy against Rheumatoid Arthritis
Source: Research (Wash D C). 2025 Sep 18;8:0844. doi: 10.34133/research.0844 (PMC12444032; doi:10.34133/research.0844)
Supplement: Supplementary 1 — Figs. S1 to S24 [file research.0844.f1.docx]

SUPPLEMENTARY MATERIALS

Supplementary Materials: Gelatin, methacrylic acid, 2-hydroxy-2-methylpropiophenone (HMPP) were purchased from Sigma-Aldrich. Methacrylate gelatin (GelMA) was synthesized using gelatin and methacrylic acid in laboratory. Silicone oil (100cs) was purchased from Shinetsu. 1,1,1,2,3,4,4,5,5,5-decafluoropentane (PFC) was purchased from Nanjing Chemlin Chemical Industry. Oxygen was purchased from Taobao. Fetal bovine serum (FBS), penicillin/streptomycin, Trypsin-EDTA solution, phosphate buffer saline was purchased from Gibco. The Live & Dead Viability/Cytotoxicity Assay Kit was purchased from Keygen Biotech Company. The Cell-Counting Kit-8 was purchased from Dojindo. The ultrasound equipment is Sonoplus 190 (Enarf-Nonius, Holland), and the ultrasound parameters applied in this research were 1.2 W cm^-2^ intensity power, 1.0 MHz central frequency, and 80% duty cycle. The Sprague-Dawley rats were purchased from the Model Animals Research Center of Nanjing University. All animal experiments were strictly in accordance with guidelines set by the Animal Ethics Committee of Drum Tower Hospital affiliated to Medical School of Nanjing University (No. 2021AE01030).

Figures S1 to S19:


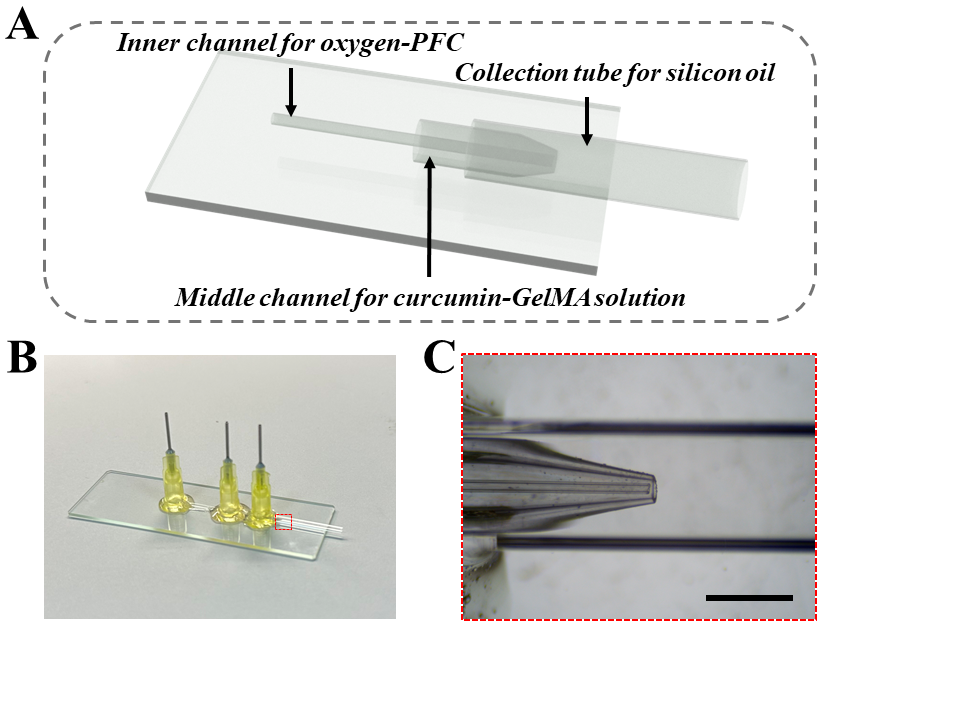


Fig. S1. Characterization of the double-emulsion microfluidic chip. (A) Schematic illustration of the microfluidic chip. (B) Digital camera image of the microfluidic chip. (B) Enlarged details of the micro-channels. Scale bar is 500 μm in (C).


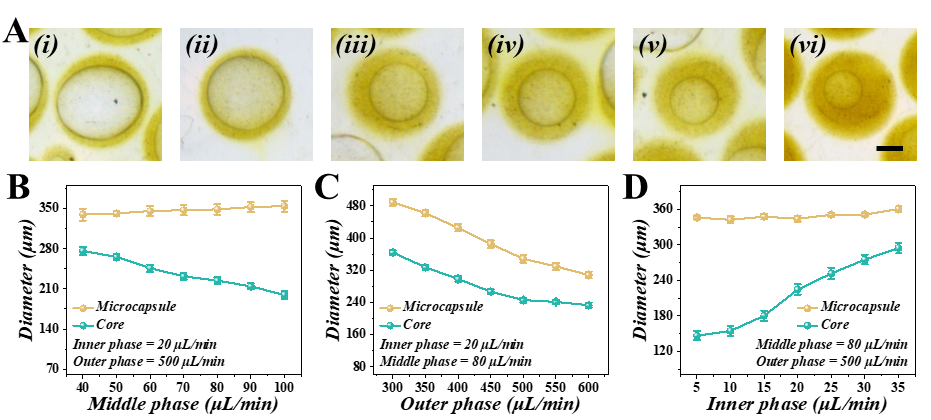


Fig. S2. Relationship between the flow rate and microcapsules’ core-total ratio. (A) Microcapsules with different core-total ratios. (B-D) Mediation of the inner, middle, and outer flow rate (n = 5). Sale bar is 100 μm in (A).


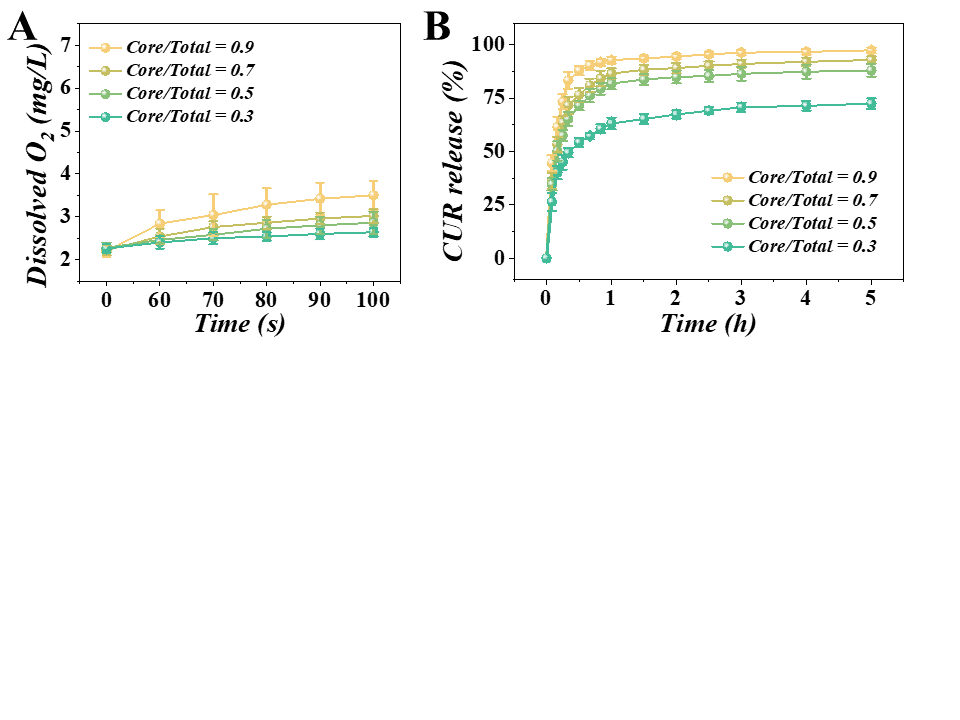


Fig. S3. Further characterization of the microcapsules. (A) Oxygen release without ultrasound stimulation (n = 5). (B) Curcumin release curves of microcapsules with different core-total ratios within 5 h (n = 5).


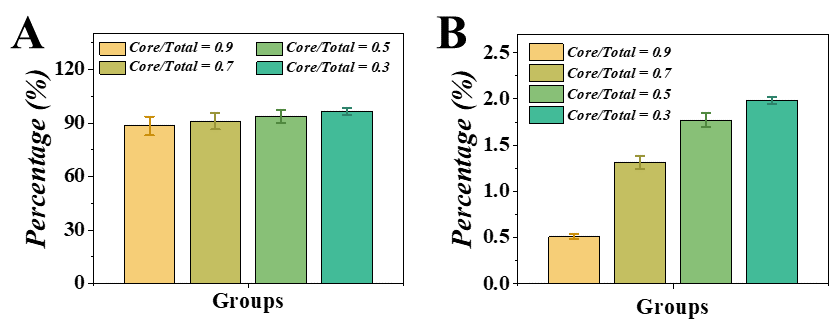


Fig. S4. Encapsulation efficiency (A) and loading capacity (B) of microcapsules with different core-total ratios (n = 5).


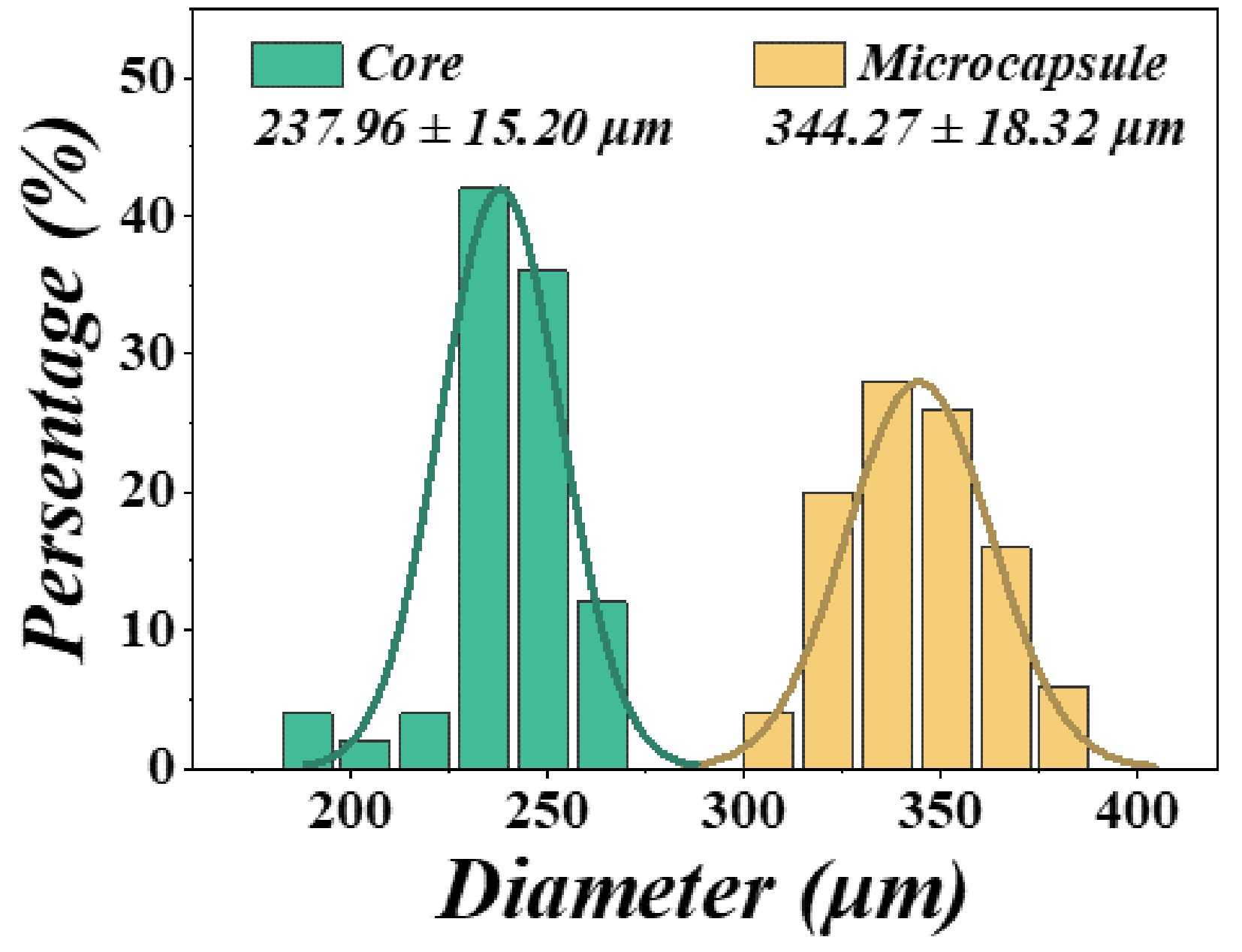


Fig. S5. Diameter distribution of the core and microcapsule diameter under 20 μL/min PFC flow rate, 80 μL/min GelMA flow rate, and 500 μL/min silicon oil flow rate (n = 50).


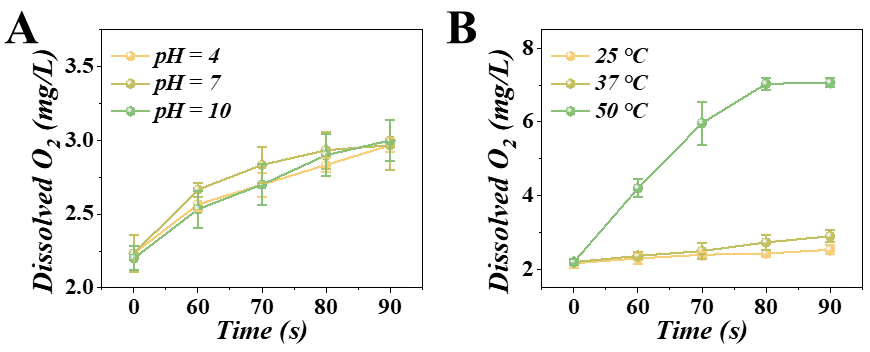


Fig. S6. Stability analyses of the microcapsules in media with different pH levels (A) and temperatures (B) (n = 3).


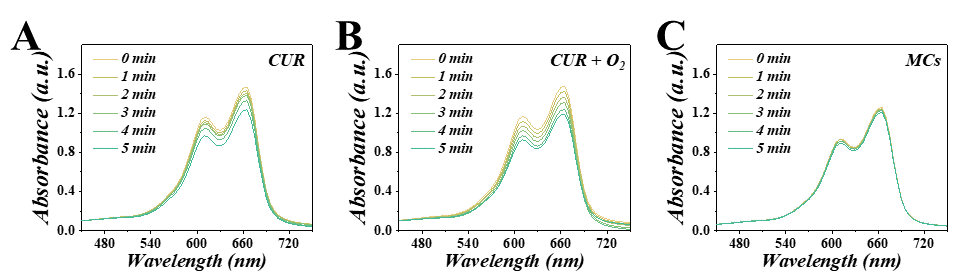


Fig. S7. Methylene blue degradation analyses of CUR group (A), CUR + O_2_ group (B), and MCs group (C).


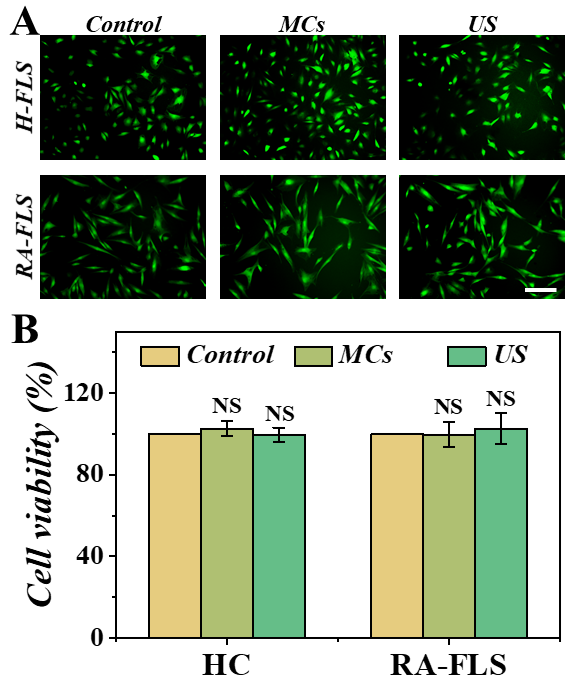


Fig. S8. Biocompatible analysis of microparticles and ultrasound. (A) Calcein AM staining of healthy and RA fibroblast-like synoviocytes. (B) CCK-8 results of healthy and RA fibroblast-like synoviocytes. Sale bar is 200 μm.


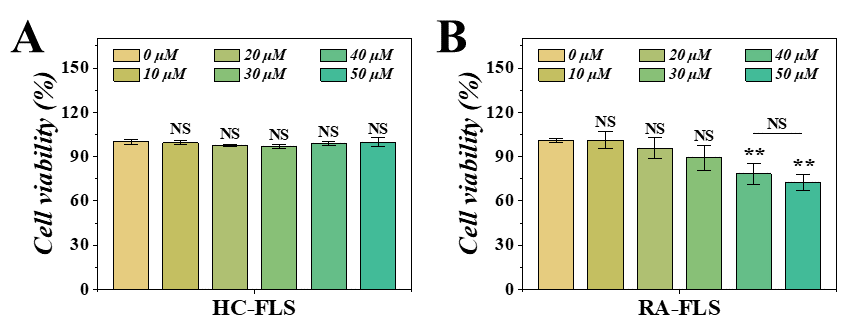


Fig. S9. CCK-8 analyses of healthy (A) and RA fibroblast-like synoviocytes (B) treated with different concentration curcumin (n = 3).


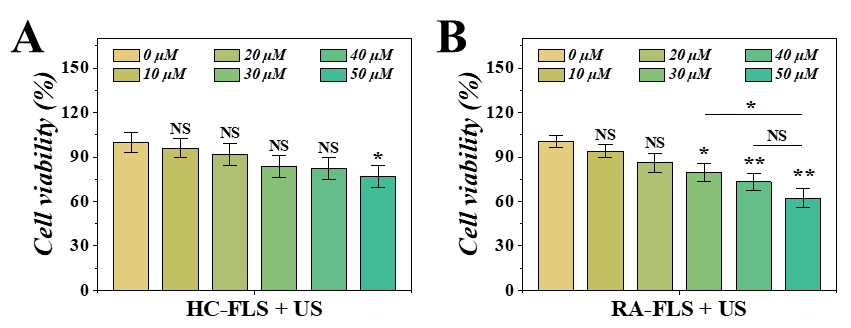


Fig. S10. CCK-8 analyses of healthy (A) and RA fibroblast-like (B) synoviocytes treated with different concentration curcumin accompanied with ultrasound stimulation (n = 3).


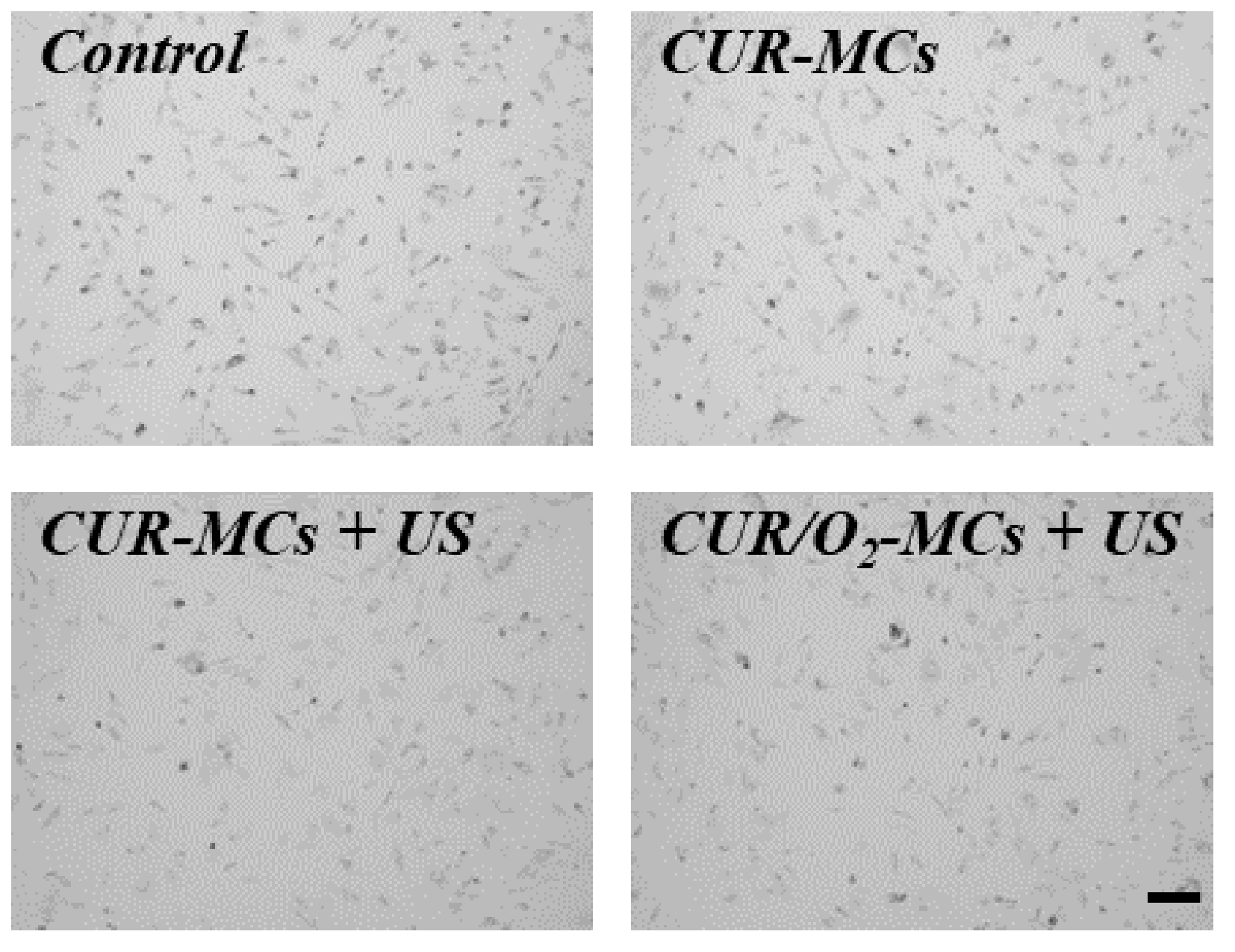


Fig. S11. Bright field images of RA-FLS in intracellular ROS analysis. Sale bar is 200 μm.


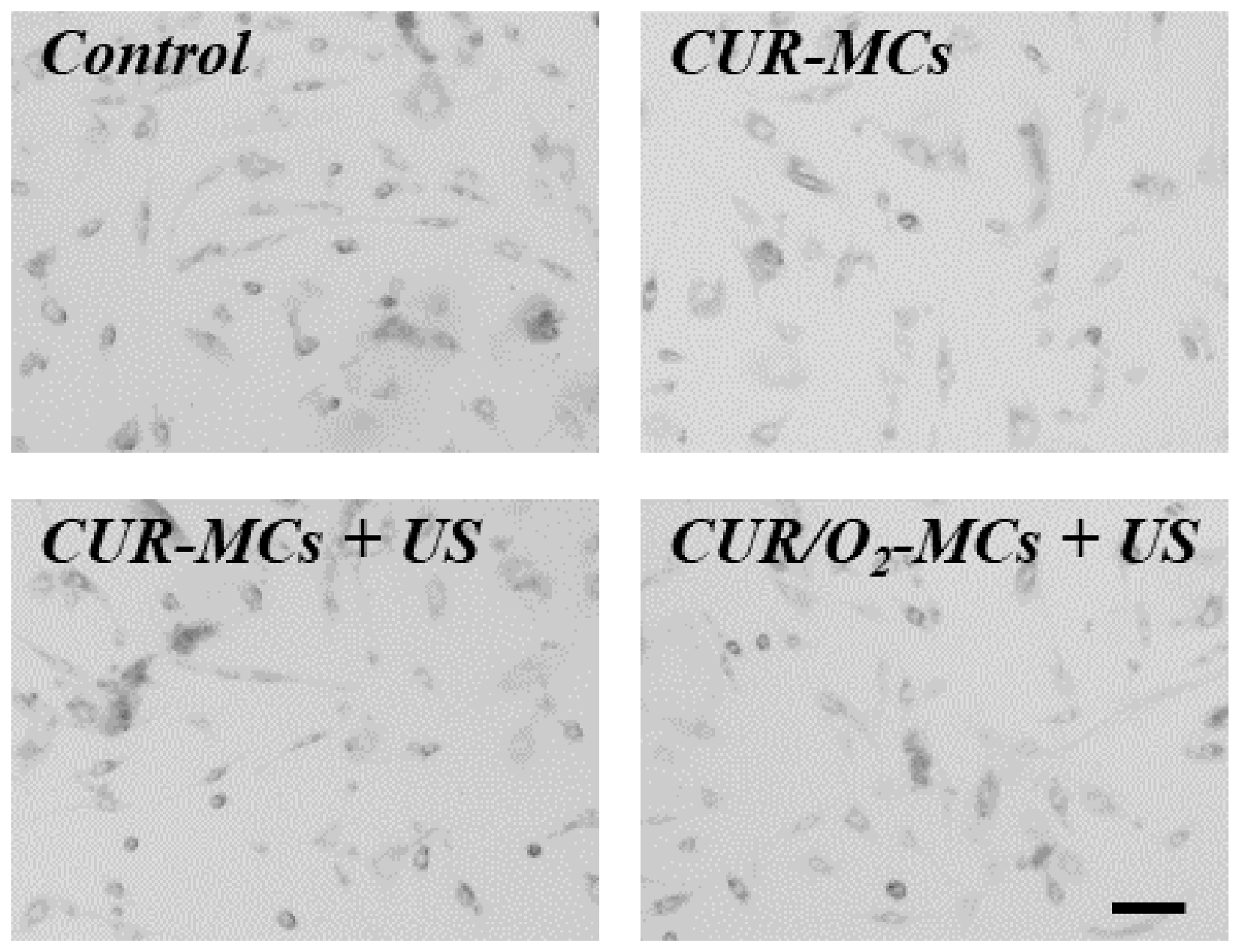


Fig. S12. Bright field images of RA-FLS in hypoxia analysis. Sale bar is 100 μm.


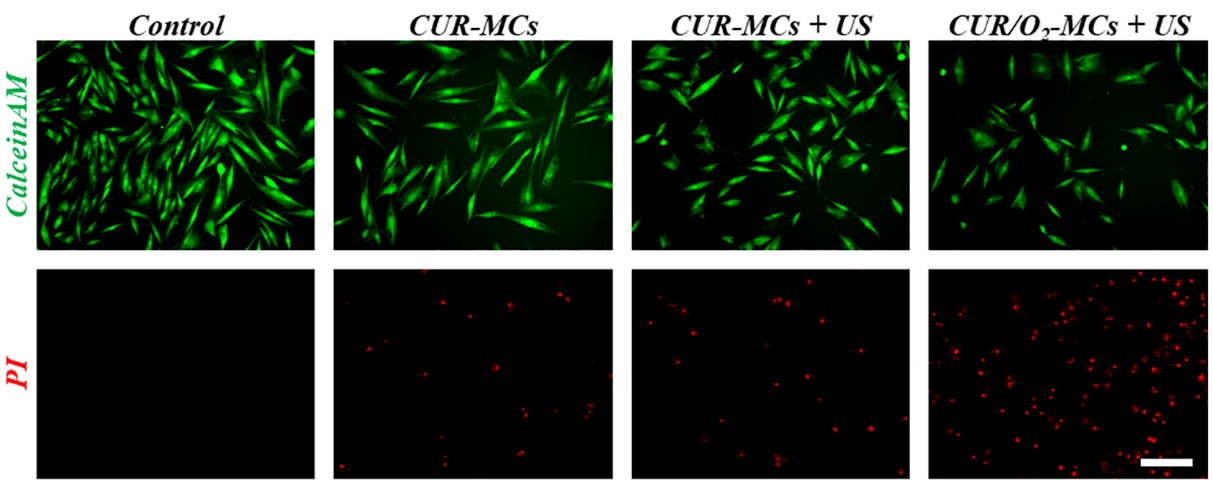


Fig. S13. Single channel fluorescence staining images of Calcein AM and PI from different groups. Sale bar is 200 μm.


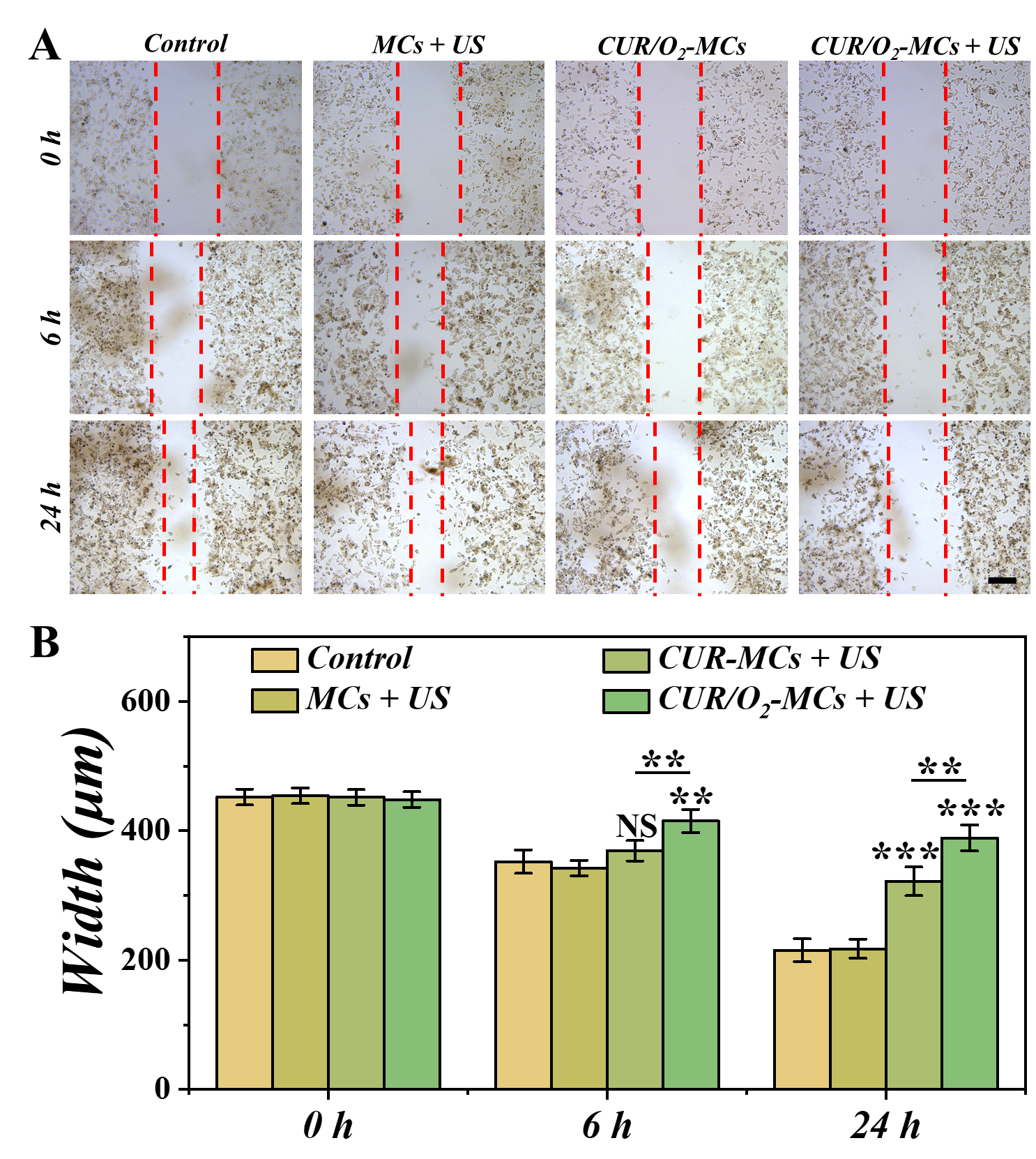


Fig. S14. Cell migration analysis of RA-FLS. (A) Photos of the RA-FLS migration at 0, 6, and 24 h. (B) Statistical analyses of the width (n = 5). Scale var is 200 μm in (A).


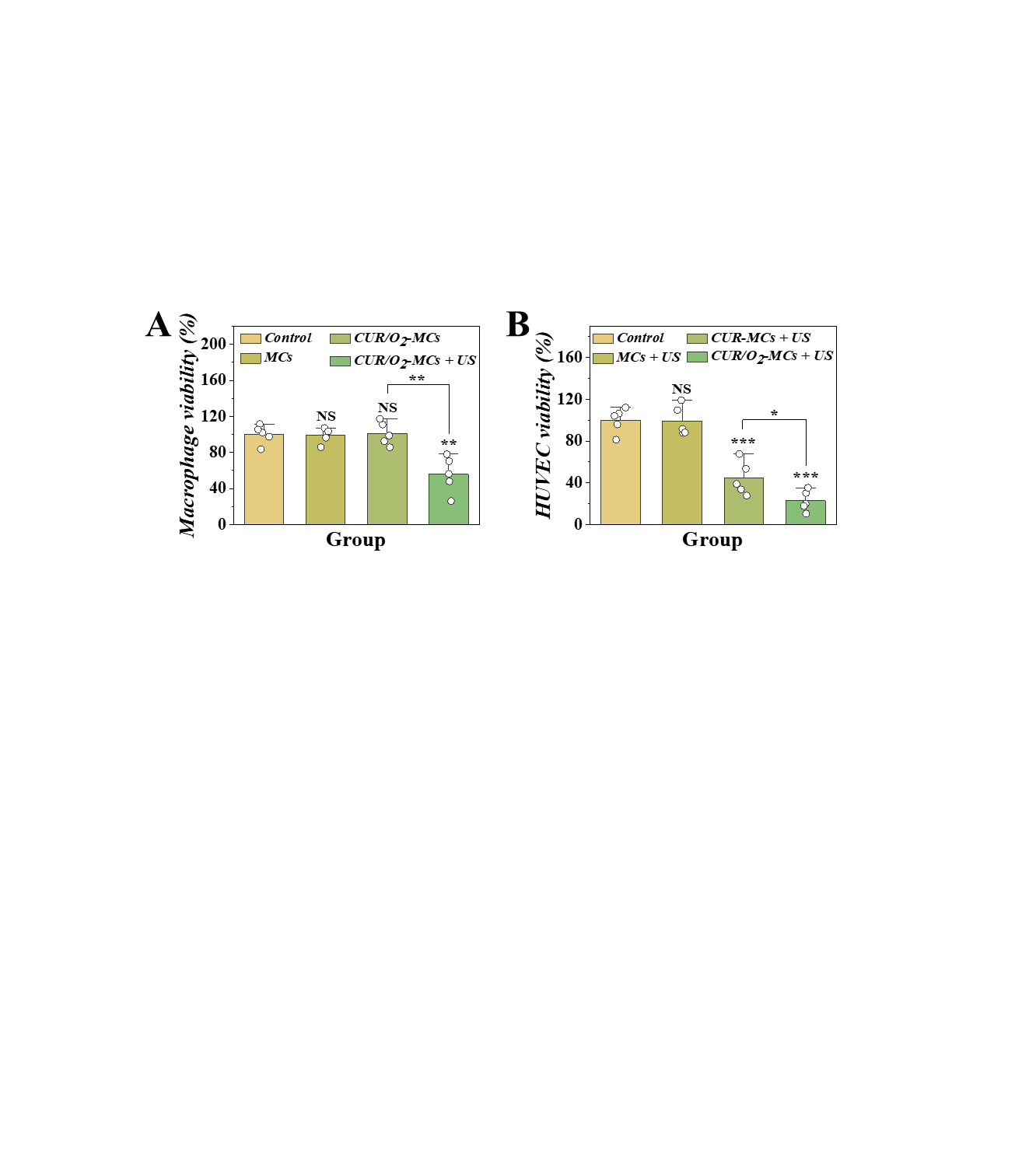


Fig. S15. Macrophage (A) and HUVEC (B) viability after sonodynamic therapy (n = 5).


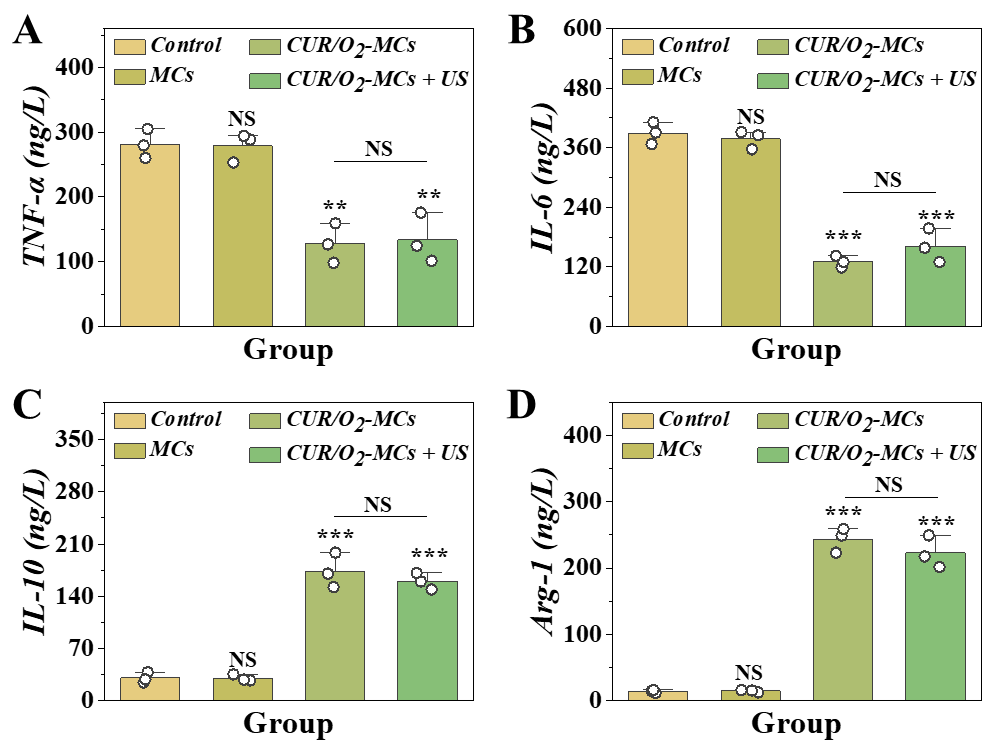


Fig. S16. Pro-inflammatory (A, B) and anti-inflammatory (C, D) cytokines detected through supernatant of RAW-264.7 cells (n = 3).


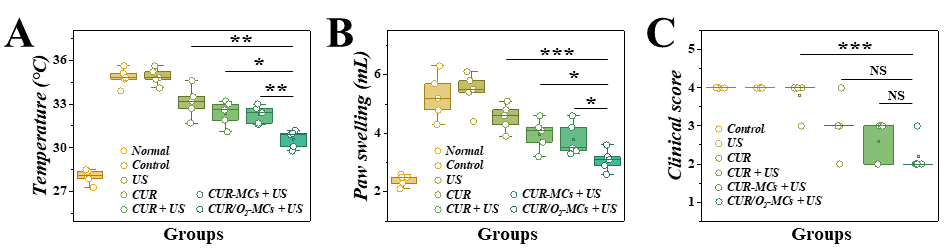


Fig. S17. Paw temperatures, paw volumes, and clinical scores from different groups at day 21 (n = 5).


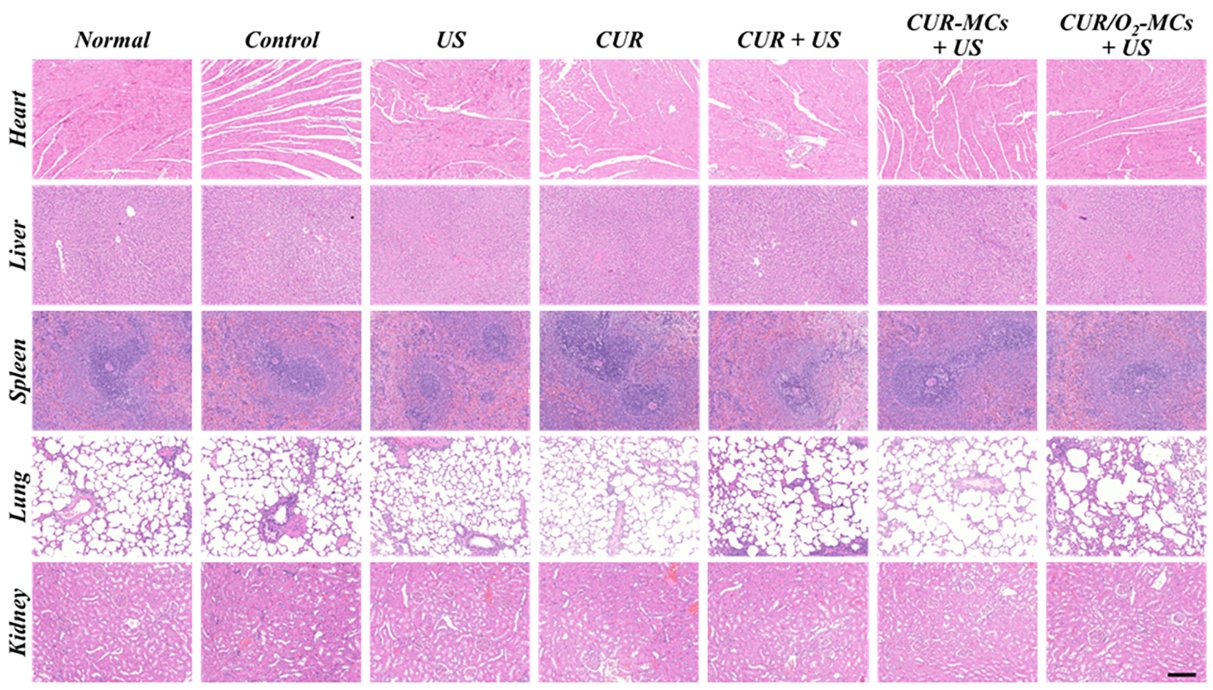


Fig. S18. HE images of main organs from different groups. Scale bar is 200 μm.


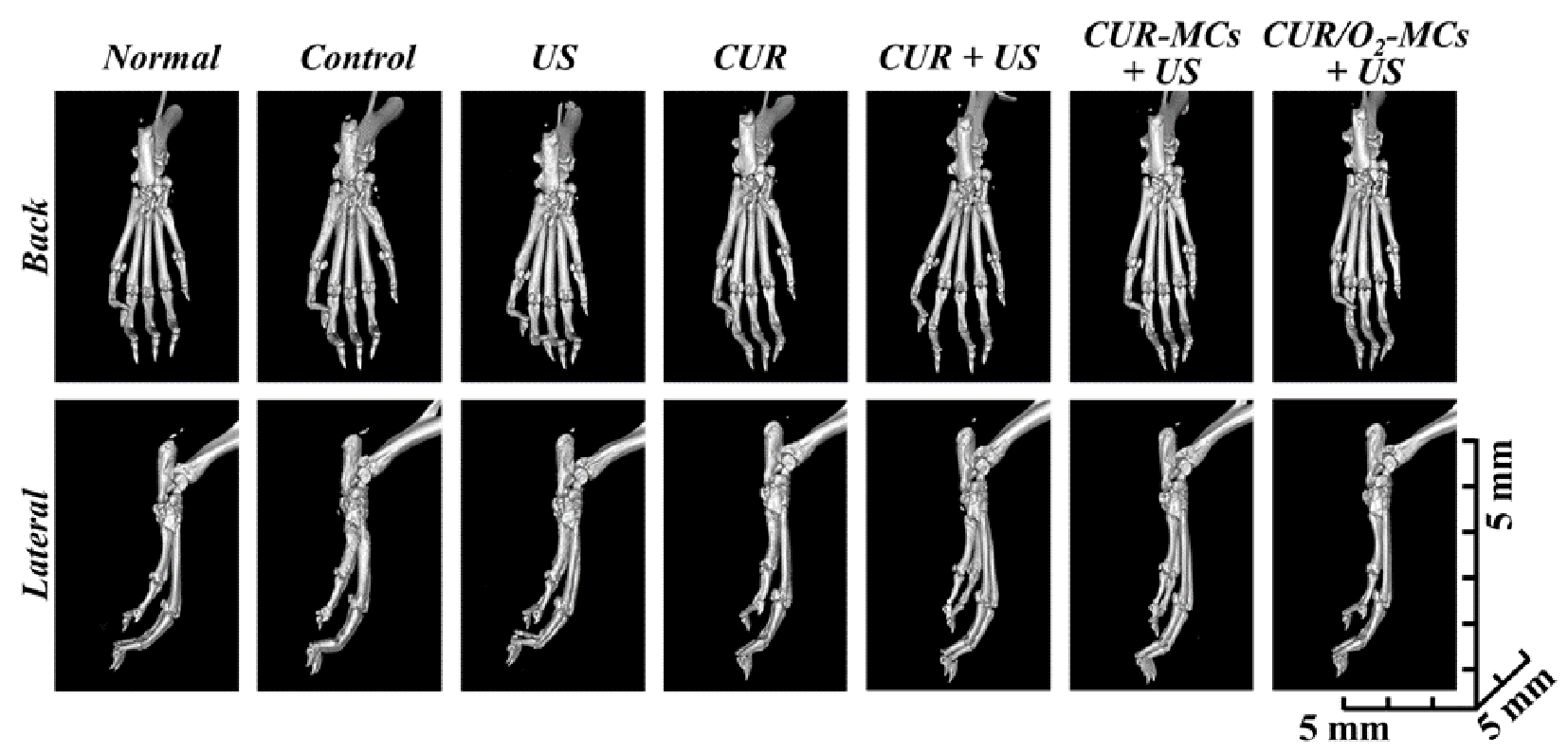


Fig. S19. Micro-CT images of the back and lateral view of paws from different groups.


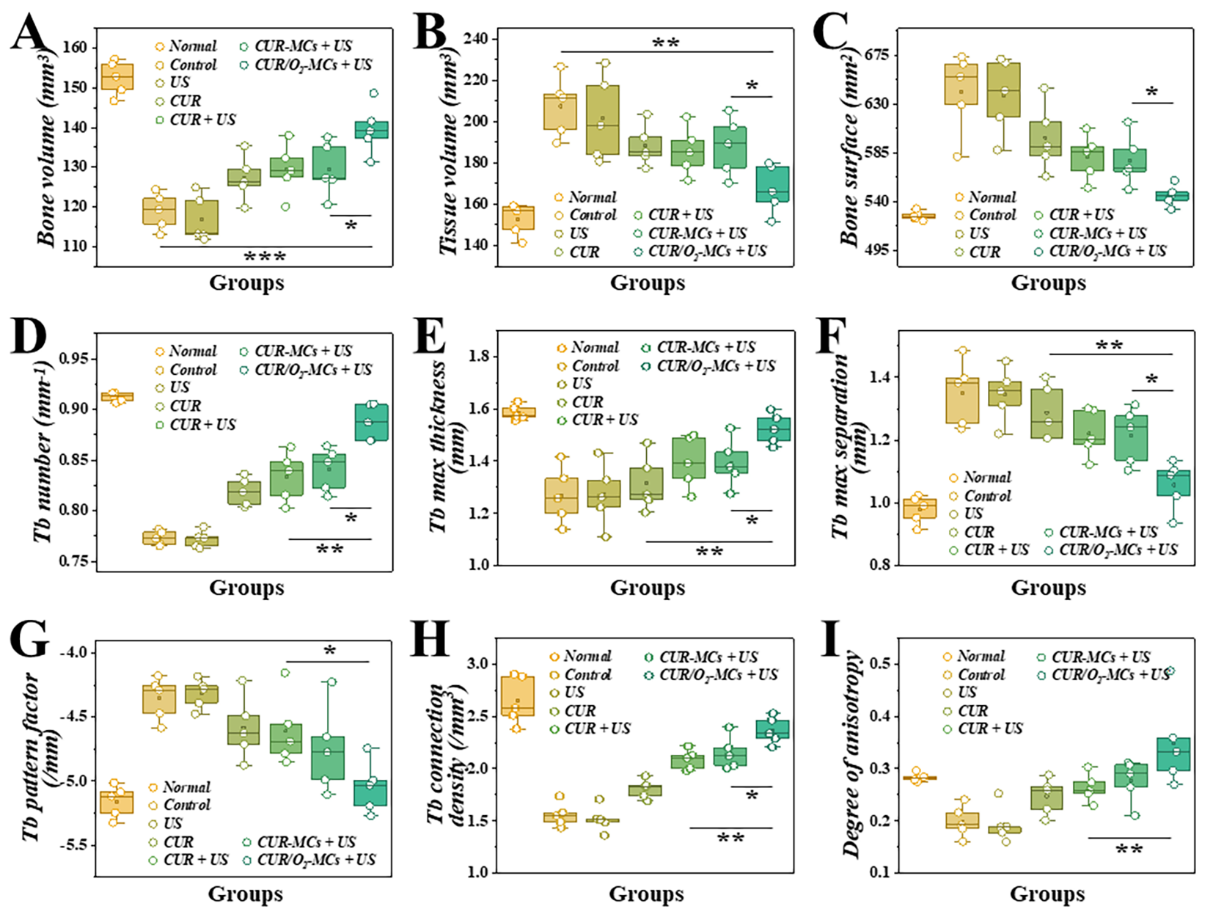


Fig. S20. Micro-CT results. (A) Bone volume. (B) Tissue volume. (C) Bone surface. (D) Trabecular (Tb) number. (E) Maximum of Tb thickness. (F) Maximum of Tb separation. (G) Tb pattern factor. (H) Tb connection density. (I) Degree of anisotropy (n = 5).


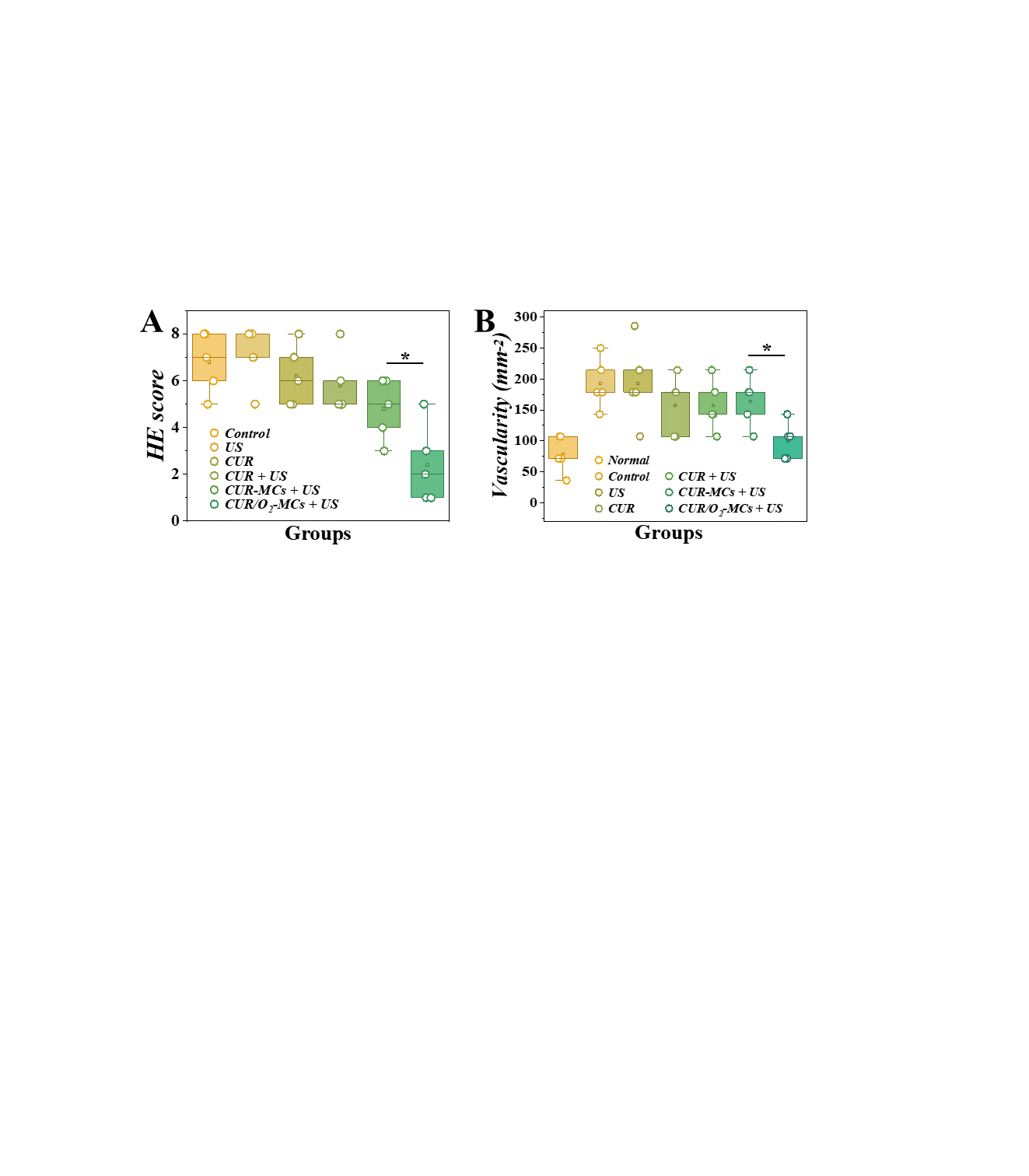


Fig. S21. HE scores of joints (A) and vascularity counting of synovial tissues (B) from different groups (n = 5).


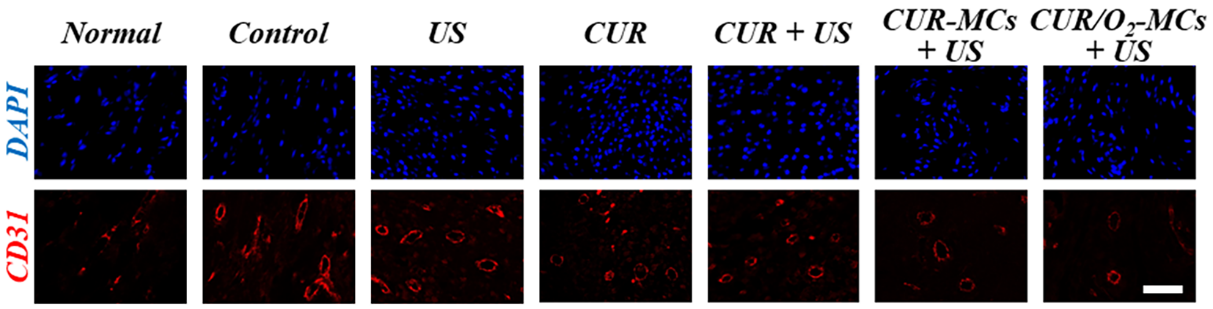


Fig. S22. Single channel fluorescence staining images of DAPI and CD31 from different groups. Scale bar is 50 μm.


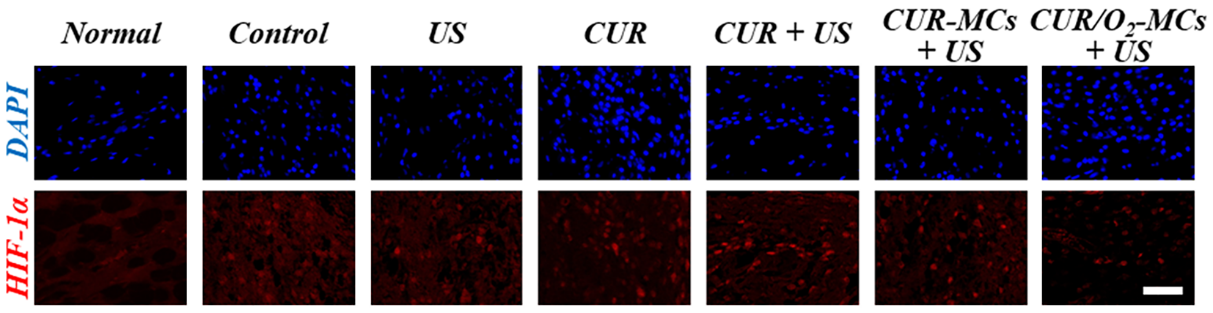


Fig. S23. Single channel fluorescence staining images of DAPI and HIF-1α from different groups. Scale bar is 50 μm.


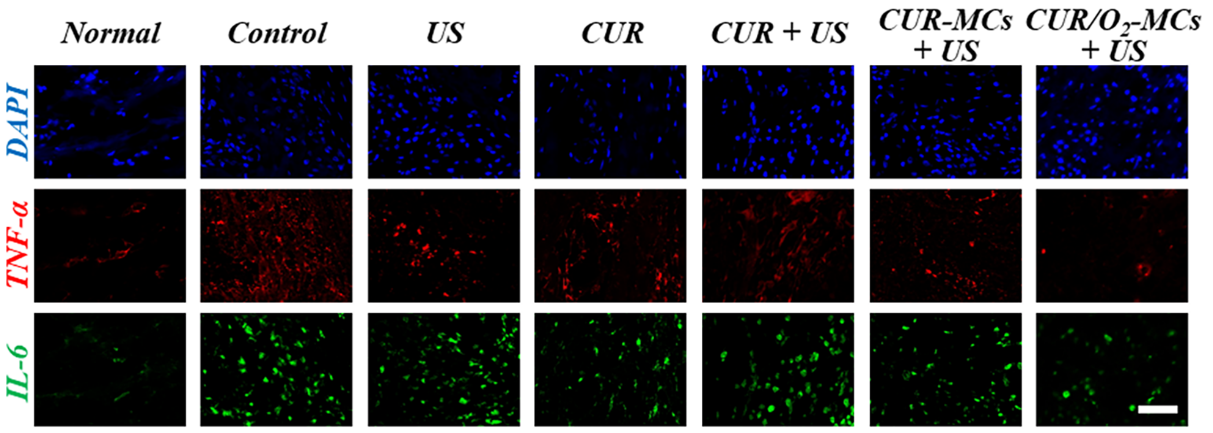


Fig. S24. Single channel fluorescence staining images of DAPI, TNF-α, and IL-6 from different groups. Scale bar is 50 μm.
